# Axially multifocal metalens for 3D volumetric photoacoustic imaging of neuromelanin in live brain organoid

**DOI:** 10.1126/sciadv.adr0654

**Published:** 2025-01-15

**Authors:** Aleksandr Barulin, Elena Barulina, Dong Kyo Oh, Yongjae Jo, Hyemi Park, Soomin Park, Hyunjun Kye, Jeesu Kim, Jinhee Yoo, Junhyung Kim, Gyusoo Bak, Yangkyu Kim, Hyunjung Kang, Yujin Park, Jong-Chan Park, Junsuk Rho, Byullee Park, Inki Kim

**Affiliations:** ^1^ Department of Biophysics, Institute of Quantum Biophysics, Sungkyunkwan University, Suwon 16419, Republic of Korea.; ^2^ Department of Intelligent Precision Healthcare Convergence, Sungkyunkwan University, Suwon 16419, Republic of Korea.; ^3^ Moscow Center for Advanced Studies, Kulakova str. 20, Moscow 123592, Russia.; ^4^ Department of Mechanical Engineering, Pohang University of Science and Technology (POSTECH), Pohang 37673, Republic of Korea.; ^5^ Department of Optics and Mechatronics Engineering, Pusan National University, Busan 46241, Republic of Korea.; ^6^ Department of MetaBioHealth, Sungkyunkwan University, Suwon 16419, Republic of Korea.; ^7^ Department of Biopharmaceutical Convergence, Sungkyunkwan University, Suwon 16419, Republic of Korea.; ^8^ Department of Chemical Engineering, Pohang University of Science and Technology (POSTECH), Pohang 37673, Republic of Korea.; ^9^ Department of Electrical Engineering, Pohang University of Science and Technology (POSTECH), Pohang 37673, Republic of Korea.; ^10^ POSCO-POSTECH-RIST Convergence Research Center for Flat Optics and Metaphotonics, Pohang 37673, Republic of Korea.; ^11^ National Institute of Nanomaterials Technology (NINT), Pohang 37673, Republic of Korea.

## Abstract

Optical resolution photoacoustic imaging of uneven samples without z-scanning is transformative for the fast analysis and diagnosis of diseases. However, current approaches to elongate the depth of field (DOF) typically imply cumbersome postprocessing procedures, bulky optical element ensembles, or substantial excitation beam side lobes. Metasurface technology allows for the phase modulation of light and the miniaturization of imaging systems to wavelength-size thickness. Here, we propose a metalens composed of submicrometer-thick titanium oxide nanopillars, which generates an elongated beam of diffraction-limited diameter with an aspect ratio of 286 and a uniform intensity throughout the DOF. The metalens enhances visualization of phantom samples with tilted surfaces compared to conventional lenses. Moreover, the volumetric imaging of neuromelanin is facilitated for depths of up to 500 micrometers within the human midbrain and forebrain organoids that are 3D biological models of human brain regions. This approach provides a miniaturized platform for neurodegenerative disease diagnosis and drug discovery.

## Teaser

Axially multifocal metalens technology enhances 3D photoacoustic imaging for rapid neuromelanin tracking in brain organoids.

## INTRODUCTION

Human brain organoids (hBOs), which are three-dimensional (3D) and miniaturized brains derived from embryonic stem cells (ESCs) or induced pluripotent stem cells, have recently gained considerable attention as unique 3D in vitro systems that allow the study of brain-related diseases or drug efficiency because they bear features of real human brain regions (*1*, *2*). Notably, flawed neuromelanin generation, owing to the loss of dopaminergic neurons in the human midbrain, is believed to correlate with the pathological mechanisms of Parkinson’s disease (PD) (*3*–*5*). However, the heterogeneity and complexity of organoids, including variations in cellular compactness, presence of necrotic cores, and distribution of protein aggregation, present notable challenges to researchers (*6*). Conventional optical imaging for organoid cross-sectional observation is a representative method, but the inherent opacity of biological tissues limits the imaging depth of standard bright-field (BF) microscopy to approximately 100 μm (*7*). This necessitates time-consuming processes such as 2D histology or 3D histology with tissue clearing methods before imaging. In this regard, the development of advanced noninvasive imaging methods without fluorescent dyes or tags is imperative for visualizing live organoids.

Label-free volumetric photoacoustic (PA) imaging technique, capable of providing deep tissue imaging without the need for external labeling of optically absorbing molecules, stands as an excellent choice for neuromelanin organoid imaging (*8*). PA imaging harnesses ultrasound waves generated by the rapid expansion and contraction of tissues upon absorbing pulsed laser light. These waves are then detected to produce images based on optical absorption contrast, offering valuable structural and functional insights into biological tissues (*9*–*11*). Neuromelanin, characterized by a robust optical absorption coefficient for visible light, proves highly conducive to observation through PA imaging (*5*, *12*, *13*). In particular, optical-resolution photoacoustic microscopy (OR-PAM) uses tightly focused light to obtain high-resolution images up to approximately 1-mm depth in biological tissue (*14*). As the axial resolution of OR-PAM depends on time-resolved ultrasound wave detection (*15*), a long depth of field (DOF) can substantially improve the OR-PAM capabilities for the volumetric imaging of samples with uneven surfaces and high penetration depths (*16*–*20*). Unlike the conventional Gaussian-beam photoacoustic microscopy (PAM), the elongated beam OR-PAM facilitates high-resolution imaging of slide-free specimens on one imaging plane, circumventing the necessity of multiple scanning steps in the axial direction. Multiple techniques to elongate the DOF for PA imaging have been previously demonstrated, such as structured illumination (*20*, *21*), contour-scanning PAM (*22*), and synthetic aperture focusing (*23*, *24*). However, their applications are associated with imaging slowdown and cumbersome image postprocessing steps. Alternatively, Bessel-beam–based PAM is similar to conventional PA imaging, with subwavelength resolutions and a long DOF (*25*–*27*). Nevertheless, Bessel beam generation is associated with substantial side lobes (*16*). Moreover, the Bessel beam intensity substantially varies within the DOF, which leads to uneven excitation of irregular specimens. Generation of a high–aspect ratio elongated beam via incident wavefront shaping provides an opportunity to minimize side lobes and maintain uniform excitation intensity of the sample along a defined axial distance (*16*, *17*, *28*). Diffractive optical elements (DOE) that are either combined with objective lenses (*16*, *17*) or used independently (*28*) have recently been demonstrated to facilitate PAM with an elongated DOF by modulating the lengths of the light propagation paths inside the unit cells of the DOE. However, one of the two studies fell short of compactness requirements due to the necessity of an objective lens, while the remaining study did not address the validation for biological tissue, posing limitations.

**Fig. 1.**
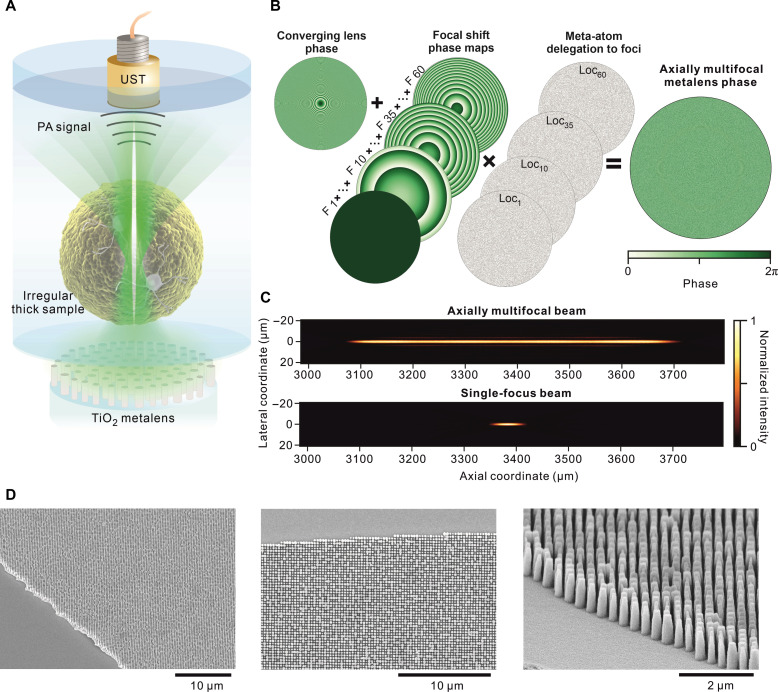
Metalens design for PAM with elongated DOF. (**A**) Schematic of the MeD-PAM designed to capture images of brain organoids with uneven surfaces. UST, ultrasound transducer. (**B**) Phase map design approach. The converging lens phase is appended to the focal shift phases that are selectively attributed to particular meta-atoms via the binary matrices generation (Loc*_k_*) to produce the final phase map. F_1_, F_10_, F_35_, and F_60_ correspond to focal shift phase maps of focus numbers. (**C**) Numerical simulations of resulting axially multifocal beam intensity profile (top) and single-focus beam intensity profile (bottom) for a NA of 0.15. (**D**) Scanning electron microscope images of the fabricated metalens structure at various magnifications and view angles.

In contrast, metalenses modulate the phase through interactions with nanoscale meta-atoms deposited on a substrate, which makes them ultracompact and truly planar photonic devices capable of controlling the properties of incident light (*29*–*31*). Moreover, metalens technology has already outperformed diffractive-lens technologies in several aspects, including high-efficiency high–numerical aperture (NA) applications, footprint reduction, tunability, and polarization sensitivity (*32*). Although metalenses theoretically exhibit unique properties owing to the precise phase control by the subwavelength-sized meta-atoms for high-resolution OR-PAM (*33*), to the best of our knowledge, their application in metalens-based elongated DOF PAM (MeD-PAM) has not been experimentally demonstrated. In this study, we propose a transmitting metalens device composed of titanium oxide nanopillars, which generates an axially multifocal beam at a 532-nm wavelength without any objective lenses (Fig. 1A). We attribute the phase values of the multiple foci distributed along the optical axis to meta-atoms in an adaptive manner and append the phase pattern of a converging lens with a defined NA of 0.15 to the phase map that corrects the elongated beam diameter and foci positions. By adjusting the phase values corresponding to the shifts of each focus, we retrieve the diffraction-limited lateral resolution. Controlling the foci positions yields an elongated beam with highly uniform intensity, diffraction-limited lateral resolution, and faint side lobes within the DOF. Common design approaches for phase and amplitude maps, including the extended Nijboer-Zernike wavefront shaping theory (*28*, *34*) and inverse design methods (*35*), are associated with cumbersome computations and limited elongated beam shape control. In contrast, the design method used in this study exhibits computational simplicity without compromising optical performance. Notably, the metasurface device generates an elongated beam from the incident plane wave without integrating an objective lens. We experimentally verify the MeD-PAM performance with elongated DOF by imaging carbon fibers and a carbon leaf tilted with respect to the glass substrate. The obtained PA imaging resolution is 2.03 μm in lateral dimensions with an elongated DOF of approximately 580 μm. In addition, we use the MeD-PAM to monitor neuromelanin granules formed in human brain organoids with a broader range of axial positions than that accessible by conventional refractive aberration-free lenses. Conventional BF microscopy visualizes neuromelanin with reduced contrast only within a shallow depth range beyond the tissue surface (*5*, *7*). Thus, the axial position information of neuromelanin inclusions cannot be determined. In contrast, MeD-PAM can quantify the neuromelanin content and follow its accumulation with maturation time within the entire live brain organoids in 3D. Thus, the significance of this work includes the following aspects: the pioneering, to the best of our knowledge, demonstration of generation of the needle beam with phase modulating high-refractive-index material metalens, straightforward design method of the single-layer phase map for the elongated beam generation, enhanced compactness and footprint over objective lenses or DOE, observation of 3D neuromelanin distribution in melanin-containing forebrain and midbrain organoids, which confirms the potential applicability of metalens technology in biological studies. Together, the axially multifocal metalens is an effective photonic device for ultracompact MeD-PAM with clinical applications in disease monitoring.

## RESULTS

### Axially multifocal metalens design

Figure 1B shows the approach used to generate the metalens phase map by forward design. The metalens is designed with a fixed NA of 0.15 and 1-mm diameter. The axially multifocal beam is composed of 60 foci distributed along the optical axis, spaced approximately by 10 μm (*d*_interfoci_) from each other. We defined the focal distances according to the following relation: *f_k_* = *f*_1_ + (*k* − 1)[*d*_interfoci_ + *V*·(*k* − 1)], where *f_k_* and *f*_1_ are the focal lengths of the *k*th focus and first focus, respectively; *k* denotes the focus number, and *V* is an optimization parameter modifying subsequent foci separation distances. The maximal separation distance is maintained considerably below the Rayleigh distance of each focus (*Z*_R_ ≈ 13.5 μm) to provide an elongated beam shape and uniform intensity along the DOF. To encode the focusing ability in the same device, the converging lens phase $\varphi_{\text{lens}}\left(x,y\right)=-2\pi /\lambda \cdot \left(\sqrt{x^{2}+y^{2}+f_{1}^{2}}-f_{1}\right)$ is included as the base for the phase map design. Then, we add the focal shift phase increments to split the foci positions throughout a designed spatial range. The focal shift phases ($\varphi_{\text{foci}}$) are given as follows (*28*)

φfoci(x,y)=∑k=1K[−πn(x2+y2)1fk−1f1/λ−πkA]·Lock(x,y) (1)

Here, *k* denotes the focus number; *K* is the total number of foci; *n* is the refractive index of the embedding medium (*n* = 1 in air); λ is the metalens working wavelength (532 nm); *A* is a coefficient that regulates the beam diameter and side lobe intensities; and Loc*_k_* is a binary matrix that adopts a value of 1 if a meta-atom is assigned to focus *k* and a value of 0 otherwise. Given that the phase map produces a single focus, further adding phase increment of focal splitting with the phase adjusters −πkA produces the following output field: $O\left(x,y,z,f_{1},\ldots ,f_{K},K\right)=\sum_{k=1}^{K}\left[F\left(x,y,z,f_{k}\right)\cdot \text{exp}\left(-i\pi \mathrm{kA}\right)\right]⊗\text{FT}\left[{\text{Loc}}_{k}\left(x,y\right)\right],$ with F(x,y,z,f) being the focus function with the focal distance *f* and FT standing for Fourier transform. $\text{FT}\left[{\text{Loc}}_{k}\left(x,y\right)\right]$ is approximated by δ(x,y)/K (*36*); therefore, the output field is approximately equal to 1K∑k=1K[F(x,y,z,fk)·exp(−iπkA)]. Hence, the output field is proportional to a summation of produced foci functions with given focal distances. The choice of Loc*_k_* matrix notably affects the resulting beam shape and intensity uniformity. We compare various approaches for generating Loc*_k_* matrices to achieve the most uniform elongated beam with reduced side lobes via numerical simulations of light propagation (Materials and Methods). A widely used approach for lateral multifocal beam generation by allocating metalens surface regions of equal area (*37*) to foci [e.g., rings (*38*, *39*) and sectors (*40*)] leads to low field uniformity within the elongated DOF, which is attributed to the interferences caused by the present grating effects within the metasurface and large number of foci (fig. S1). Attributing each meta-atom to a particular focus with a uniform random distribution prevents ripples or substantial intensity variations throughout the beam DOF. The parameter *V* represents the spacing change between successive focal points. Correct choice of this parameter enables to either increase or decrease interfocal distance for farther foci and therefore modulates the axial intensity gradient within the DOF (fig. S2). We account for the natural enlargement of axial focal waists at longer focal lengths and optimized *V* parameter to 15 nm which yielded uniformly distributed intensity along the entire elongated DOF. Various random functions that fix random states yield apparently identical side lobe intensities or beam diameters (fig. S3) making the method rather insensitive to random number generator algorithms. An *A* parameter plays a key role in controlling the beam diameter via modulating optical interferences occurring among the foci (*36*). By increasing the value of *A*, the beam diameter can be drastically reduced to approximately the diffraction limit; however, this is achieved at the cost of increasing the side lobe intensities (fig. S4). We find that a value of 0.14 for *A* produces nearly diffraction-limited performance together with rather low side lobes, especially at the edges of the DOF. The values below 0.1 would result to slightly lower side lobes and wider beam diameter; therefore, *A* parameters around 0.1 would corroborate well with the observations found in prior arts (*16*) and would provide a nearly diffraction-limited beam diameter with modest side lobes. The axial intensity profile shows that the elongated beam DOF is approximately 13.5 times longer than the DOF of a simulated conventional lens of equivalent NA (Fig. 1C). Although the metalens point spread function (PSF) might resemble that of the Bessel beam, the angles of the wavelets are not fixed and depend on the meta-atom delegation to the focus number, whereas axicons redirect collimated beam wavelets at a fixed refraction angle. A Bessel beam of similar length can be numerically produced by an annular phase mask (fig. S5). However, the axial intensity profiles would yield substantial oscillations leading to nonuniform molecule excitation throughout the DOF. Unlike the approach of axial multiple focusing, axicons are deprived of fine-tuning the axial intensity profile. Moreover, the side lobe intensities of the simulated Bessel beam always exceed 16%, while those of the multifocal beam are at the level of 9%.

### Fabrication and optical characterization of metalens

We use circular nanopillars of titanium oxide particle composite to match the target phase pattern. Titanium oxide exhibits low losses at a working wavelength of 532 nm, whereas 900-nm-high nanopillars with a pitch of 300 nm provide full-2π range phase control through diameter tuning from 90 to 255 nm (fig. S6). The nanopillars of selected diameter and height obey the Nyquist sampling criterion (unit cell size < NA·λ/2) (*41*) and exhibit high near-field transmission. Moreover, the unit cell size below 330 nm yields no abrupt phase variations that enable fine phase control (fig. S7). After finding a meta-atom arrangement that replicates the target phase map, the axially multifocal metalens is fabricated. First, a silicon master mold is fabricated by electron beam lithography (EBL), and subsequently, a soft mold of the complementary pattern is created (fig. S8). The titanium oxide medium for the meta-atoms is prepared using titanium oxide particles embedded in a polymer resin coated on the soft mold and cured under pressure against a glass slide. The fabrication of the metalens with a 1-mm diameter is completed after removing the soft mold. The pillar-shaped meta-atoms exhibit well-defined circular nanopillar geometries with fixed pitch sizes (Fig. 1D).

**Fig. 2.**
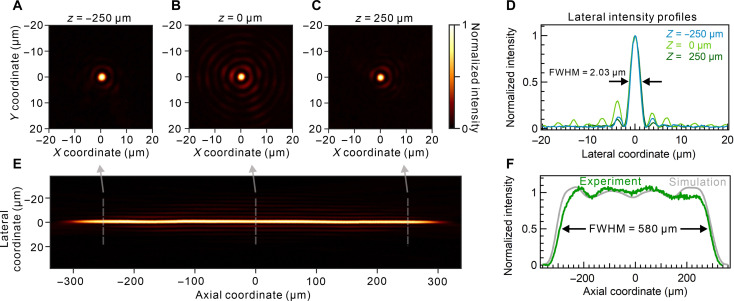
Experimental characterization of metalens PSF. (**A** to **C**) Lateral focal spot intensity profiles at axial coordinates of *z* = −250, 0, and 250 μm, respectively. Axial coordinate *z* = 0 μm corresponds to the central plane of the elongated beam. (**D**) Horizontal cuts of the intensity profiles shown in (A) to (C). (**E**) Axial intensity profile of the elongated beam. (**F**) Axial intensity profile cut obtained from the numerical simulations and experimental data.

The experimental characterization of the PSF is conducted on a transmission microscope setup with a 532-nm continuous-wave laser source (fig. S9) by sending a collimated laser beam toward the metalens and subsequently imaging the PSF at multiple planes (see Materials and Methods). The results confirm a constant beam diameter throughout the elongated axial beam length (Fig. 2, A to C). The average lateral intensity full width at half maximum (FWHM) equals 2.03 μm and remains nearly constant over at least 500 μm (Fig. 2D). Figure 2E presents the elongated beam shape of the metalens with high axial intensity profile uniformity and weak side lobes. The SD of the intensity over an axial distance of 500 μm within the DOF does not exceed 5%, which coincides with the results of known approaches based on the extended Nijboer-Zernike theory (*17*, *28*). The axial intensity FWHM amounts to 580 μm in remarkable agreement with the simulated data (Fig. 2F). The focusing efficiency of the metalens amounts to 20%, which is determined to be the ratio of the laser power in the central focus spot area and laser power incident onto the metalens. The modest value of focusing efficiency is attributed to several factors including (i) fabrication defects, (ii) reduction of the absolute of value of power in the main lobe due to the presence of phase regulator term (*A*), (iii) present Rayleigh scattering at the meta-atoms due to their stochastic refractive index variation within the metasurface (*42*), and (iv) large field elongation leading to reduction of the efficiency value as compared to a single-focus lens. Nonetheless, this method enables highly uniform field profile within DOF, tight focusing and focusing efficiency comparable to meta-axicons (*27*). The numerical optimization appears to be computationally simple to enable nearly any beam aspect ratios (the ratio of the beam axial size to its lateral size). The background noise stemming from nonmodulated and scattered light may adversely affect the PA measurements; however, its substantial influence is noticed only in the proximity to the metalens interface, while the background level at the distance of the elongated beam generation is negligible, as confirmed by the optical measurements of PSF (Fig. 2F). It is worth noting that the power delivered to the main lobes of the Bessel beams may be at the level of 5% (*36*), while the reported focusing efficiency of the multifocal metalens substantially exceeds that value.

### Performance demonstration of MeD-PAM

**Fig. 3.**
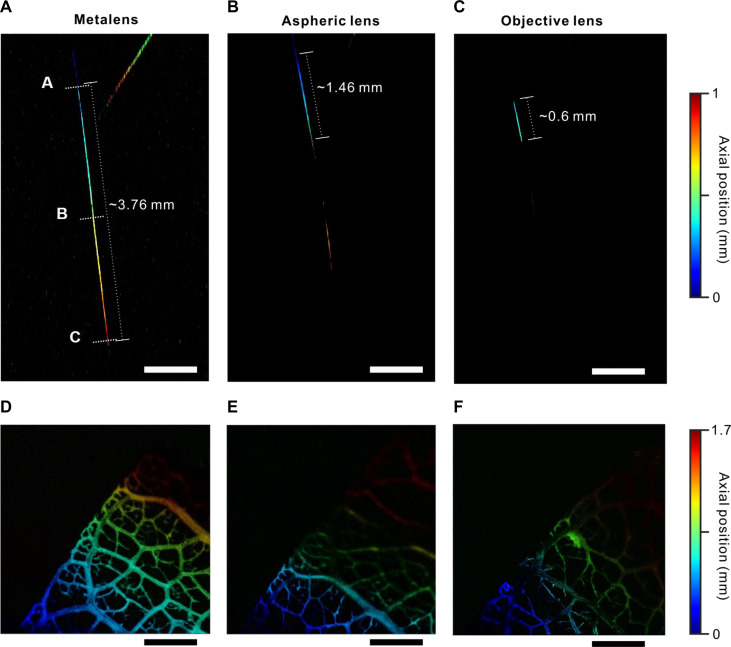
Phantom sample elongated DOF PA imaging. (**A** to **C**) Depth-encoded PA images of two axially tilted carbon fibers acquired using (A) metalens, (B) aspheric lens, and (C) objective lens. The letters shown in (A) corresponds to line profiles shown in fig. S13. The distance values indicated correspond to the length of left fiber that is visible on the PA images. (**D** to **F**) Depth-encoded PA images of axially inclined carbon leaf acquired using (D) metalens, (E) aspheric lens, and (F) objective lens. Scale bars, 500 μm.

We develop a home-built transmission-mode MeD-PAM (fig. S10). To demonstrate the benefit of MeD-PAM, we obtain PA images using the metalens and conventional lenses, an aspheric lens with an effective NA of 0.12, and an objective lens with an effective NA of 0.19. To provide a correct comparison of the imaging performance, we maintain equal power density incident on the sample for all three lens options. According to the experimental data shown in fig. S11, the DOF of the metalens (580 μm) appears to be 3.8 times longer than that of the aspheric lens (152 μm) and 27.1 times longer than that of the objective lens (21.4 μm). Given the inverse square dependence of DOF on NA (*43*), the objective lens exhibits DOF close to the lower bound set by the diffraction limit. The aspheric lens DOF is expected to equal 49 μm; however, possible aberrations lead to the geometrical optical DOF increase. Notably, the lateral resolution of the metalens exceeds that of the aspheric lens, which makes it a promising device for high-resolution PAM imaging. Carbon fibers represent a phantom sample with optical absorption contrast. Tautened carbon fibers have been fixed at different angles and heights yielding axial surface gradient that would be challenging to observe with shallow DOF PAM. Brief PA data acquisition and reconstruction methods can be found in Materials and Methods. First, we evaluate the axial profile of the PA signal by sliding the elongated beam through the horizontal carbon fiber in the water medium. The PA signal is recorded at different distances between the metalens and carbon fiber. The PA signal profile on the axial coordinate corroborates the optical measurements (fig. S12). Next, aiming to manifest the advantage of the metalens functionalities for compact PA imaging, we prepare a phantom sample of two carbon fibers vertically inclined at 4° 45′ and 9° 30′ with respect to the substrate plane. We demonstrate that the MeD-PAM provides a clear image of an axially tilted carbon fiber over a substantially longer in-plane distance when compared to single-focus refractive lenses (Fig. 3, A to C). The laser power is adjusted to equalize the power density incident onto the sample for each lens. Owing to the time-resolved ultrasound wave detection, we could decode the depth information from the carbon fiber data (see Materials and Methods). The color-encoded depth information of the carbon fibers confirms their axial tilt. The elongated DOF PA images show discernible signals from the carbon fibers over a distance of at least 2.6 times longer than that of the aspherical lens and 6.3 times longer than that of the objective lens. Moreover, MeD-PAM visualizes two carbon fibers during a single plane scanning, whereas conventional PAM fails to visualize both, owing to the substantial axial position mismatch between the two carbon fibers. The carbon fiber PA image acquired by the aspheric lens exhibits certain nonnegligible signal lobes at higher axial positions (Fig. 3B), which can be attributed to the substantial light intensity ripples beyond the focal plane (fig. S11). We calculated line profiles at three different lines (A, B, and C) in Fig. 3A to confirm that the lateral resolution of MeD-PAM is well maintained at different depths of the carbon fiber (fig. S13). The FWHM of the three-line profiles is 11.5 ± 0.27 μm, which aligns well with the known thickness of the carbon fiber, approximately 5 to 10 μm. In addition, we set a carbon leaf with an axial position gradient along the lateral diagonal as a second phantom sample (Fig. 3, D to F). We observe that the metalens ensures a rather sharp PA image of carbon fibers within the leaf in a field of view window of approximately 3 mm with precise axial position determination. In contrast, the PA images acquired by the conventional lenses exhibit sharp leaf focusing only within the limited area, whereas the leaf parts outside the single-focus lens DOF appear blurred and yield a lower PA signal.

### 3D high-resolution and deep-tissue neuromelanin PA imaging in live brain organoids

**Fig. 4.**
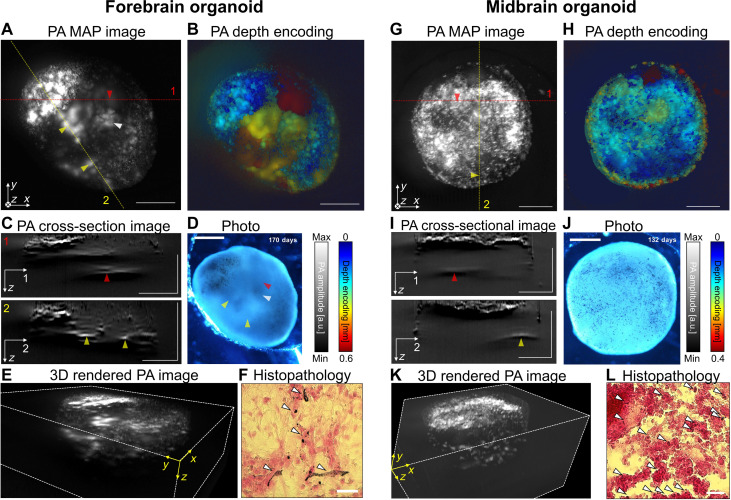
Volumetric PA imaging of neuromelanin in melanin-containing forebrain and midbrain organoids with axially multifocal metalens. (**A**) PA MAP image of forebrain organoid grown for 170 days. (**B**) Depth-encoded PA image of forebrain organoid with colors representing axial positions of neuromelanin. (**C**) PA cross-sectional image of forebrain organoid. The upper cross-sectional image corresponds to the red dashed line in (A). Bottom cross-sectional PA image is acquired along the yellow dashed line in (A). (**D**) BF image of forebrain organoid. The four arrows in (A) and (D) point to the same location. (**E**) 3D rendered PA image of forebrain organoid. (**F**) Fontana-Masson staining of forebrain organoid. Arrows indicate the black pigments in the forebrain organoid. (**G**) PA MAP image of midbrain organoid grown for 132 days. (**H**) Depth-encoded PA image of midbrain organoid with colors representing the axial positions of neuromelanin. (**I**) PA cross-sectional image. The upper cross-sectional image corresponds to the red dashed line in (A). Bottom cross-sectional PA image is acquired along the yellow dashed line in (A). (**J**) BF image of midbrain organoid. (**K**) 3D rendered PA image of midbrain organoid. (**L**) Fontana-Masson staining of midbrain organoid. Arrows indicate the black pigments in the midbrain organoid. Scale bars, 500 μm. a.u., arbitrary units.

A guided self-organizing method for the generation of brain organoids is used for the cultivation of human-induced pluripotent stem cells (hiPSCs), according to a previous study, with minor modification (see Materials and Methods and fig. S14) (*2*). PA images of neuromelanin distribution in the 3D forebrain and midbrain organoids are photoacoustically acquired and analyzed for the effective application of MeD-PAM to biological samples (Fig. 4). First, we prepare a melanin-containing forebrain organoid to visualize the inner part of neuromelanin signals, speculating that partially expressed dopaminergic neurons (fig. S15) could also produce the inner part of neuromelanin, although the majority of the mature forebrain neurons are glutamatergic neurons (*44*). We confirm that melanin-producing cell types [SRY-box transcription factor 10 (SOX10), a biomarker for neural crest precursor cells; dopamine transporter (DAT), a biomarker for dopaminergic neurons] are expressed for both melanin-containing forebrain organoids and midbrain organoids (fig. S16). The PA maximum amplitude projection (PA MAP) image in Fig. 4A is acquired from the forebrain organoid. The four arrows in Fig. 4 (A and D) point to the same location. Black pigments of neuromelanin in the forebrain organoid are observed in the bright-field (BF) image (Fig. 4D). However, except for the upper left corner, neuromelanin within the organoid is blurred, especially at the locations indicated by the four arrows. Neuromelanin is predominantly formed inside the forebrain organoid and is difficult to observe using general optical methods (*5*). In contrast, the PA MAP image reveals details of the neuromelanin signals throughout the organoid. In particular, the positions indicated by the four arrows exhibit a pronounced difference compared to what is observed in the BF image. This demonstrates the effective delineation of the 3D organoid interior by the elongated DOF in PAM. Figure 4B presents a depth-encoded PA image with different colors representing neuromelanin signals at different depths, wherein blue corresponds to signals near the organoid surface and red indicates neuromelanin signals in deeper tissue. Depth-encoded images may be affected by unavoidable background signals. However, these images serve as a supplementary tool that intuitively illustrates the depth distribution of melanin. For a more detailed analysis, depth cross-sectional images of the 3D PA image are examined (Fig. 4C). The first cross section follows the red dashed line in Fig. 4A, with the red arrow indicating neuromelanin PA signals at a depth of 0.5 mm within the organoid (#1 in Fig. 4C). This signal is faintly observed in the image, making depth estimation challenging. The second cross-sectional PA image acquired along the yellow dashed line in Fig. 4A reveals the dynamic distribution of neuromelanin at deeper depths within the organoid (#2 in Fig. 4C). The upper left primarily represents neuromelanin close to the surface, and the two yellow arrows indicate neuromelanin signals at approximately 0.31-mm depth, which are also faintly observed in the image. Figure 4E showcases a 3D-rendered forebrain organoid, allowing simultaneous visualization of neuromelanin signals at various depths (movie S1). We conduct a histopathology study to confirm that the signals identified in the forebrain PA and BF images originate from neuromelanin. Fontana-Masson staining results show the partial accumulation of neuromelanin as black pigments (Fig. 4F and fig. S15B). Furthermore, the correlative imaging of PA and Fontana-Masson staining supports that PA contrast originates from the neuromelanin and demonstrates the specificity of MeD-PAM for neuromelanin (fig. S17). For more information, validation of dopaminergic neuronal gene (dopamine transporter, DAT) expression and neural crest cell gene expression (SOX10) in the forebrain organoid is performed through immunohistochemistry (figs. S15 and S16).

Next, we conduct PA imaging of midbrain organoids, a variant of brain organoids known for their substantial accumulation of neuromelanin, given that dopaminergic neurons constitute a major neuronal cell population within them (*3*–*5*). The PA MAP image presented in Fig. 4G is obtained from the midbrain organoid depicted in Fig. 4J. In comparison to the forebrain organoid, the PA MAP and BF images of the midbrain organoid reveal a more widespread distribution of neuromelanin across the entire surface. In addition, the depth-encoded PA midbrain organoid image in Fig. 4H indicates that neuromelanin is predominantly formed near the organoid surface (mostly blur and light blue colors). For a more comprehensive analysis, cross-sectional images of the 3D PA image are examined (Fig. 4I). The initial cross-sectional analysis traces along the red dashed line in Fig. 4G; the red arrow denotes neuromelanin PA signals at a depth of 0.44 mm within the organoid (#1 in Fig. 4I). In addition, this cross-sectional image reveals a uniform distribution of neuromelanin across the organoid surface. Notably, deeper neuromelanin PA signals indicated by the red arrow are indiscernible in the accompanying image. The second cross-sectional PA image, obtained along the yellow dashed line in Fig. 4G, also illustrates the uniform distribution of neuromelanin near the surface (#2 in Fig. 4I). The yellow arrow points to neuromelanin signals at a depth of approximately 0.37 mm, which also remain invisible in the image. Figure 4K presents a 3D-rendered forebrain organoid, enabling simultaneous visualization of neuromelanin signals at various depths (movie S2). Our verification indicates that most of the mature neurons in the midbrain organoids are dopaminergic neurons (DAT^+^/MAP2^+^) (fig. S15), and an abundant accumulation of black pigments is observed in the Fontana-Masson–stained image (Fig. 4L). We perform PA imaging of the forebrain and midbrain organoids with the axially multifocal metalens and conventional lenses (fig. S18). The aspheric lens and objective lens produce PA images of similar lateral resolutions and qualities; however, they are unable to visualize neuromelanin signatures at depths greater than 200 μm beyond the glass-organoid interface.

### Quantification of neuromelanin accumulation in live brain organoids using MeD-PAM

**Fig. 5.**
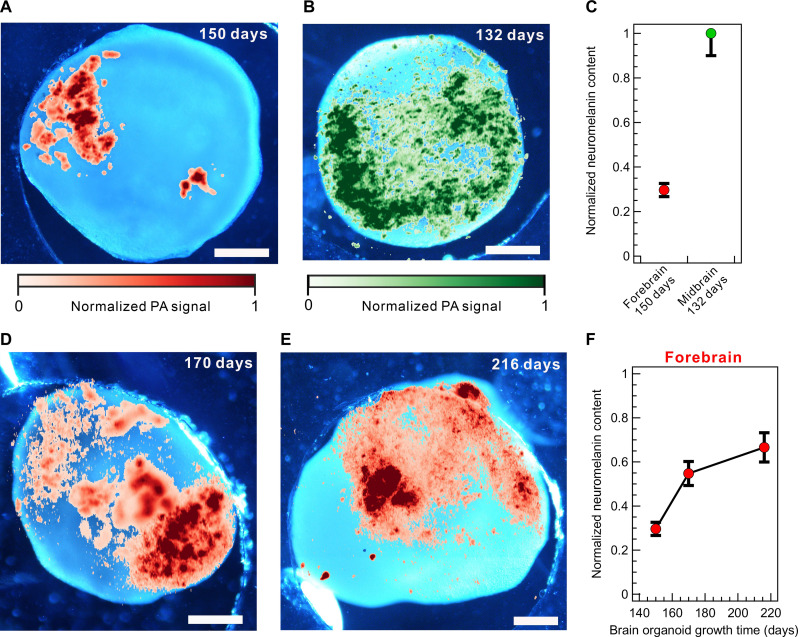
Neuromelanin content characterization of various melanin-containing forebrain and midbrain organoids with MeD-PAM. (**A**) Overlaid BF and PA images of forebrain organoid on day 150. (**B**) Overlaid BF and PA images of midbrain organoids on day 132. (**C**) Normalized neuromelanin content of brain organoids from (A) and (B). The neuromelanin content is evaluated by summing up the PA image pixels exhibiting signals above the background noise, with high fidelity. Error bars correspond to neuromelanin content variations caused by reducing the threshold value within the SD of background noise. (**D** and **E**) Overlaid BF and PA images of forebrain organoids on days 170 and 216, respectively. (**F**) Normalized neuromelanin content of brain organoids from (A), (D), and (E). Scale bars, 500 μm.

Furthermore, we leverage the capabilities of MeD-PAM to quantitatively analyze the neuromelanin content within brain organoids. We enhance the clarity by overlaying the acquired MeD-PAM images with BF images (Fig. 5). Initially, we examine the forebrain and midbrain organoids with similar growth times. The results reveal a substantial difference, with the midbrain organoid containing substantially higher levels of neuromelanin than the forebrain organoid (Fig. 5, A and B). For quantitative analysis, the neuromelanin content is determined to be the sum of PA image pixels exhibiting signals exceeding three times the SD of the background noise. The neuromelanin content is normalized to that of the midbrain organoid, which exhibits the highest neuromelanin content among the studied variants. On day 132, the midbrain organoid contains 3.4 times more neuromelanin than the forebrain organoid at a similar maturation stage (on day 150) (Fig. 5C). Notably, in the overlaid images, neuromelanin is uniformly present in the midbrain organoid; however, it is partially present in dense clusters in the forebrain organoid. Immunohistochemistry and Fontana-Masson data confirm the formation of dopaminergic neurons and neuromelanin granules in a similar fashion (fig. S15), which is assumed to be the source of the observed neuromelanin granule patterns in the MeD-PAM (*3*). Last, we characterize a series of forebrain organoids of different age groups to track the progression of melanogenesis (Fig. 5, A, D, and E). Neuromelanin granules are observed to gradually increase from 150 to 216 days of maturation time (Fig. 5F). Therefore, the MeD-PAM provides a compact imaging system facilitating quantitative screening of melanogenesis in entire live brain organoids.

## DISCUSSION

In this study, we design a titanium oxide metalens that generates an elongated beam with a high aspect ratio, uniform intensity distribution within the DOF, and weak side lobes. Notably, the metalens exhibits 3.8 times longer DOF and improved lateral resolution, compared to a commercial aspheric lens. The unique application of such light manipulation for MeD-PAM provides unique opportunities to generate 3D distributions of analytes exhibiting absorption contrast at 532 nm. The proposed truly planar metalens device has submicrometer thickness and well-controlled unit structure shapes and generates an elongated beam with the propagation phase modulation only. The MeD-PAM acquires high-resolution images through point-by-point scanning, and a smaller metalens allows for a more compact system as compared to axicons or objective lenses with DOE which represents a key advantage of our study. The intensity variations throughout the DOF inferior of 5% make the system ideal for even excitation of irregular specimens within a large axial range. Potentially, the tunability of metasurface properties, as a function of polarization, wavelength, and orbital angular momentum (*29*, *30*, *45*–*47*), can pave the way for multifunctional MeD-PAM. Also, the MeD-PAM is designed to have a transmission-mode structure, which shows sufficient performance in acquiring PA signals in an in vitro imaging environment. MeD-PAM is designed with an OR-PAM scheme, enabling the acquisition of PA signals at high resolution from biological tissues up to approximately 1 mm in depth (*14*). Therefore, MeD-PAM is well-suited in terms of imaging depth and resolution for observing these organoids. However, for larger organoids that grow to 2 to 3 mm in size, the performance of MeD-PAM may be somewhat limited. In the future iterations of the system, this can be addressed by adjusting the system, such as increasing the optical focus size and light intensity (*48*).

The selection of the 532-nm wavelength is based on the unique optical properties of melanin in the visible and near-infrared regions. Specifically, the absorption coefficient of melanin is substantially higher than that of other biomolecules, including hemoglobin, in this spectral region. While in typical tissues, the melanin signal may overlap with the hemoglobin signal, this is not a concern in our study, as the hBOs used are bloodless, eliminating any potential interference from hemoglobin (*5*, *49*). Berezhnoi *et al.* (*49*) demonstrate that melanin exhibits a strong and background-free signal across a spectral range extending from 488 to 900 nm. This includes the 532-nm wavelength, which we found to be effective for achieving clear and specific imaging of neuromelanin within the organoids. In addition, while the maximum penetration depth for green light in standard tissues is typically limited to approximately 1.5 mm due to hemoglobin absorption, our hBOs, which do not contain hemoglobin, allow for greater penetration depths. Specifically, at 650 nm, higher penetration can be achieved, and at near-infrared wavelengths, up to 3-mm penetration depth is possible without notable loss of contrast or resolution.

Although MeD-PAM is focused on melanin detection, the following considerations can enhance its practicality for application to various organoids (e.g., vascularized organoids, cardiac organoids, cancer organoids, etc.) in the future. First, multispectral PA imaging has the potential to improve the specificities of neuromelanin and hemoglobin, which can be achieved by generating multiple wavelengths using stimulated Raman shifting (*50*). Specifically, by applying multispectral PA imaging, it is possible to distinguish between a low-absorbing molecule at high local concentrations and a high-absorbing molecule at low concentrations, which would be difficult to differentiate using single-wavelength PAM. Second, real-time imaging and analysis could be highly effective in observing responses such as drug delivery in organoids. This can be realized by using an optical scanning-based high-speed PA microscope (*51*–*53*).

In general, the use of a Gaussian beam results in a very shallow focal depth, making it challenging to acquire images over a large area at once when there are substantial height variations in the sample. This limitation arises as the optical focus must be adjusted to bring each area into focus before imaging can continue. However, the extended focal depth of MeD-PAM enables the acquisition of a relatively large field of view in a single scan, even if the sample surface has irregular height variations. This capability notably reduces the need for refocusing and enhances the efficiency of imaging over uneven samples. Moreover, the metalens enables effective visualization of neuromelanin in human brain organoids with cellular resolutions in the axial position range up to 500 μm, which exceeds the capabilities of conventional Gaussian beam PAM. The axial resolution of MeD-PAM is determined to be approximately 90 μm (Materials and Methods). This study represents clear identification of the distribution of neuromelanin, but for more precise analysis, a higher-frequency ultrasound transducer could be used to achieve high-resolution axial resolution. Also, we obtain PA images of neuromelanin in organoids using ultrasound transducers with frequencies of 5, 20, and 40 MHz and compared the PA B-scan images (fig. S19). In the low-frequency 5-MHz PA B-scan, the reverberation is substantially stronger compared to the 20- and 40-MHz ultrasound transducers, which negatively affects the axial resolution. However, no notable difference is observed between the 20- and 40-MHz transducers. The signal-to-noise ratio (SNR) values obtained from the melanin of the organoid PA images from Fig. 4 (A and G) are 43.9 and 45.8 dB, respectively. The calculation of SNR (dB) follows the formula $\text{SNR}\;\left(\text{dB}\right)=20\times \left[{\text{log}}_{10}(\text{mean}\left(\text{signal}\right)/\text{std}\left(\text{noise}\right)\right]$, where std represents the SD. The SDs of the noise for these measurements are 63.7 and 84.1, respectively. According to this calculation, MeD-PAM demonstrates a high signal amplitude relative to noise.

Axicons could, in principle, provide imaging of brain organoid neuromelanin distribution as well; however, additional postprocessing deconvolution steps would be needed to diminish the influence of larger side lobes and retrieve correct 3D distribution of the target analyte amount (*25*, *54*, *55*). Of course, in the case of melanin-containing forebrain organoids, the partial presence of neuromelanin pigments may not necessarily be originated solely to partial-expressed dopaminergic neurons, as we confirmed the expression of neural crest precursor cells (SOX10^+^) in the organoids. Given that both neuronal cells and melanocytes share their common origin from neural crest cells, this observation could also be possible due to their aging processes (*56*).

We successfully monitor the melanogenesis in live organoids at various maturation stages and determine the distribution of neuromelanin content and its location in the organoid volumes. Since neuromelanin is selected as the target substance due to its key association with the pathology of PD, where the loss of dopaminergic neurons in the substantia nigra is closely correlated with decreased neuromelanin levels, we believe that our system can effectively monitor the progressive neurodegeneration observed in PD. Thus, our results pave the way for fast and portable systems for enhanced diagnostics tools and drug efficiency screening platforms based on the PAM of slide-free biological samples.

## MATERIALS AND METHODS

### Design of metalens

TiO_2_ was selected as the meta-atom material with a high refractive index and no loss at a wavelength of 532 nm (*30*). The simulated data of phase and transmittance were generated using Ansys Lumerical FDTD and based on the ellipsometrically measured refractive index of titanium oxide nanoparticle–embedded resin. The meta-atom dimensions were contained within the feasible range for fabrication, with a unit cell size of 300 nm. The target phase data were generated via numerical simulations using a Rayleigh-Sommerfeld propagator. A plane wave was used as the incident light source. After generating the optimal target phase profile, brute force search was used to find the optimal titanium oxide nanopillar diameters for the final metalens device. The generated realistic phase profile was fed to the Rayleigh-Sommerfeld propagator to simulate the expected PSF. An ideal single-focus lens PSF with working distance equivalent to that of the metalens was simulated using the same method following the lens phase equation: $\varphi_{\text{lens}}\left(x,y\right)=-2\pi /\lambda \left(\sqrt{x^{2}+y^{2}+f^{2}}-f\right)$, where λ is the laser wavelength and *f* denotes the lens focal length.

The meta-axicon is simulated using a Rayleigh-Sommerfeld propagator with the unit cell size of 300 nm. The phase equation of the axicon has been set to (*27*): $\varphi_{\text{axicon}}\left(x,y\right)=2\pi -2\pi /\lambda \cdot \sqrt{x^{2}+y^{2}}\cdot \text{NA}$, where NA equals 0.15.

### Metalens fabrication

The metalens was fabricated via the nanoimprint lithography process (*57*). The master mold for the multifocal metalens was fabricated using a high-resolution EBL system on a Si substrate. First, a photoresist (Microchem, 495 PMMA A6, MicroChem) was coated with 400-nm thickness and baked at 180°C for 5 min on the Si substrate. After exposing an optimized metalens design on the photoresist layer using the EBL system (ELS-BODEN, Elionix), the exposed area was developed using a 1:3 methyl isobutyl ketone (MIBK)/isopropyl alcohol solution. Next, a 40-nm-thick Cr layer was vertically deposited on the patterned area using an electron beam evaporation system (KVE-ENS4004, Korea Vacuum Tech Co. Ltd.) for a successful lift-off process. After removing the Cr-patterned photoresist area using acetone, the remaining Cr layer acted as a mask layer for selective Si etching. Then, dry etching (silicon/metal hybrid etcher, DMS Co. Ltd.) was performed to create 900-nm-thick Si nanostructures. The master mold for the multifocal metalens was obtained by removing the residual Cr masks using a Cr etchant (CR-7).

To make the soft mold for the high-resolution TiO_2_ nanoparticle embedded resin (nano-PER) nanoimprint lithography process, a hard polydimethylsiloxane (PDMS) (*h*-PDMS)/PDMS bilayer was adopted. First, the *h*-PDMS solution was prepared by mixing 3.4 g of vinylmethyl copolymers (VDT-731, Gelest), 18 μl of platinum catalyst (SIP6831.2, Gelest), 0.1 g of the modulator (2,4,6,8-tetramethyl-2,4,6,8-tetravinylcyclotetrasiloxane, Sigma-Aldrich), 2 g of toluene, and 1 g of siloxane-based silane reducing agent (HMS-301, Gelest). The *h*-PDMS solution was coated onto the master mold at 1000 rpm for 60 s using a spin coater and baked at 70°C for 2 hours. Then, a mixture with a 10:1 weight ratio of PDMS (Sylgard 184 A, Dow Corning) and its curing agent (Sylgard 184 B, Dow Corning) was poured on the baked *h*-PDMS layer and cured at 80°C for 2 hours. The fabricated *h*-PDMS/PDMS bilayer soft mold was detached from the master mold and coated via vaporization of a fluorosurfactant [(tridecafluoro-1,1,2,2-tetrahydrooctyl) trichlorosilane, Sigma-Aldrich] at 130°C for 5 min to reduce the surface energy of the soft mold.

The TiO_2_ nano-PER was prepared by mixing TiO_2_ nanoparticles dispersed in MIBK [DT-TIOA-30MIBK (N30), Ditto Technology], monomer (dipentaerythritol penta-/hexaacrylate, Sigma-Aldrich), photoinitiator (1-hydroxycyclohexyl phenyl ketone, Sigma-Aldrich), and MIBK solvent (MIBK, Duksan General Science). The mixing ratio was controlled to achieve a weight ratio of 4 wt % for TiO_2_ nanoparticles, 0.7 wt % for the monomer, and 0.3 wt % for the photoinitiator.

The TiO_2_ nano-PER of 0.4 ml was uniformly coated on the fabricated soft mold using a spin coater at 2000 rpm for 60 s. After the soft mold absorbed the solvent of the TiO_2_ nano-PER, the TiO_2_ nano-PER layer was pressed between the soft mold and SiO_2_ substrate at 2 bar for 60 s and cured by ultraviolet light at 8 mW/cm^2^ for 15 min. Last, the fully cured multifocal metalens was successfully transferred onto a SiO_2_ substrate by removing the soft mold.

### Metalens optical characterization

The elongated beam metalens PSF was evaluated using a microscope setup. A 532-nm wavelength collimated laser beam was guided toward the metalens with a beam size slightly larger than the diameter of the metalens. The incident beam must be well collimated, as focusing the incident light can substantially alter the elongated beam DOF and diameter otherwise. The beam focused by the metalens was visualized by an imaging system that included an Olympus UPLFLN 20X objective lens (NA = 0.5) and a Lumenera Infinity 2-1R charge-coupled device camera. The objective lens was mounted on top of a motorized stage to capture images at multiple planes. We executed a *z* directional focal profile to accurately measure the elongated beam shape and DOF. To conduct PSF measurements along the full elongated beam length, 360 images of the lateral beam dimensions were captured at intervals of 2 μm along the optical axis. The PSF measurements of the aspheric lens and objective lens of 0.6 NA were conducted using the same microscope setup. The effective NA of the conventional lenses were computed as ${\text{NA}}_{\text{eff}}=\text{sin}\left(\text{arctan}\left(r_{\text{beam}}/f\right)\right)$, where $r_{\text{beam}}$ is the radius of the incident beam, set to 1 mm, and *f* denotes the focal length of the lens. The size of the metalens was slightly smaller than the incident beam; therefore, the effective NA corresponded well with the nominal value.

### PA imaging settings

The excitation source was a 532-nm nanosecond laser (Photonics Industries International Inc., DX-Air cooled nanosecond laser, DX-532-2) with a working pulse repetition frequency of 20 kHz and a pulse width of 6 ns. The laser energy was adjusted by a half-wave plate polarizing beam splitter (VA5-532/M, Thorlabs Inc.). The laser beam was collimated with a plano-convex lens of focal length 500 mm (LA1908-A-ML, Thorlabs Inc.), and the collimated laser beam diameter was 2 mm. In the case of the OR-PAM with metalens, the laser beam diameter was reduced to 1 mm using a ring-actuated iris diaphragm (SM1D12D, Thorlabs Inc.). The axial positions of the metalens, aspherical lens, and objective lens were varied by moving the side-actuated 6.5-mm travel translation stage (MS1S/M, Thorlabs Inc.). The laser beam was focused on the sample immersed in a deionized water tank made of glass. The pulsed laser illumination energy was ~15 mJ/cm^2^ at 532 nm, which is below the American National Standards Institute safety limit for biological safety standards (*58*). The PA signal was collected using a planar high-frequency ultrasound transducer with a −6-dB center frequency of 19 MHz, a focal distance of 11.6 mm (15.7 μs), and a bandwidth of 69% (fig. S20). The lateral resolution obtained from the edge spread function of blade was 2.3 μm (fig. S21, A and B). The axial resolution measured based on the PA B-scan image of carbon fiber was 90 μm (fig. S21, C and D). The xyz translation stage (PT3/M, Thorlabs Inc.) was used for aligning the ultrasound transducer immersed in deionized water. The motorized xy scanning stage (8MTF, Standa) was used for scanning the image in the *x* and *y* directions by moving the glass petri dish. The PA signal was received using a dual pulser/receiver with a bandwidth of 500 MHz (DPR500, JSR Corporation) in the transmission mode through an ultrasound transducer. The receiving analog information was converted into a digital form by a computer with a 12-bit waveform at a 500 MS/s real-time sampling rate digitizer (ATS9352, Alazar technologies Inc.). A custom software was used for scanning using the motorized stage and saving A- and B-scans. The conventional refractive lenses used for PA imaging were an aspheric lens with an effective focal length of 6.24 mm (A110TM-A, Thorlabs Inc.) and an objective lens LUCPlanFL 40×/0.60 Ph2 (Olympus). The B-scans were postprocessed with a custom Python code that retrieved the PA intensity images and decoded the depth/axial position information. The speed of sound was set to 1540 m/s to convert the signal time delay to the axial position. The median filter of a window size of 2 × 2 pixels was applied to depth image maps to provide smooth RGB (red, green, blue) colors.

### PA image acquisition and reconstruction

PA signals were acquired by scanning in the x and y directions, capturing PA data at each position (fig. S22A). During fast scanning along the *x* axis, a B-scan dataset was obtained and stored, while slow scanning along the *y* axis was used to acquire and store 3D volume data. The PA image reconstruction was performed after the entire 3D volume data had been acquired (fig. S22B). To remove noise and high-frequency components in each B-scan dataset, Wiener and lowpass filters were applied. The filtered 3D data were then represented as a 2D grayscale PA image through maximum amplitude projection along the *z* axis and as a 2D depth-encoded PA image through depth encoding.

### Generation and characterization of brain organoids

To generate melanin-containing human forebrain organoids, a guided self-organizing method was used to cultivate hiPSCs (BIONi010-C, K3P53; The European Bank for induced pluripotent stem cells) according to our previous report, with minor modifications (*2*). Briefly, for the melanin-containing forebrain organoids, high-quality undifferentiated hiPSCs, maintained on ESC-qualified Matrigel-coated cell culture plates, were detached using ReLeSR. The detached colonies were dissociated into single cells and harvested in AggreWell embryoid body (EB) formation medium supplements with Y-27632. On day 0, the cells were seeded into AggreWell800 24-well plates. On day 1, the culture medium transitioned to the EB formation medium to initialize the self-organization process. From day 2 to day 5, the culture medium underwent bidaily refreshment with Dulbecco’s modified Eagle’s medium (DMEM)/F12-based medium containing SMAD signaling inhibitors such as 0.1 mM 2-mercaptoethanol, dorsomorphin, and SB-431542. On day 6, all organoids were cultured in a neuronal medium composed of neurobasal-A medium, B-27 supplement minus vitamin A, penicillin (100 U/ml) and streptomycin (100 μg/ml), GlutaMAX, and 0.5% basement matrigel membrane matrix. Between day 7 and day 24, medium changes were performed using the neuronal differentiation medium. Between days 25 and 42, the medium was changed every other day by the neuronal maturation medium for neuronal maturation. After day 43, only the neuronal medium underwent replacement every 4 days for maintenance with minimized plate shaking. For midbrain organoids, the protocol mentioned in a previous report was modified and applied (*3*). First, EBs were generated similar to the method implemented in the case of the forebrain and were seeded into ultralow-attachment 96-well plates (7007, Corning) filled with 50 μl of neural induction medium (IM) containing a mixture of DMEM/F12 (2645233, Thermo Fisher Scientific) and neurobasal medium (2661481, Thermo Fisher Scientific), supplemented with N2 supplement (17502048, Thermo Fisher Scientific), B27 without vitamin A (2596532, Thermo Fisher Scientific), GlutaMAX (35050061, Gibco), MEM Non-Essential Amino Acids Solution (MEM NEAA, 11140, Thermo Fisher Scientific), and β-mercaptoethanol (21985023, Gibco), along with heparin (H3149, Sigma-Aldrich), SB431542 (1614, Tocris), Noggin (CYT-475, Prospec), CHIR99021 (4423, Tocris), ROCK inhibitor Y27632 (Calbiochem), and growth factor reduced (GFR) Matrigel. On day 7, 100 μl of IM supplemented with SHH-C25II (464-SH, R&D Systems) and FGF8 (423-F8, R&D Systems) per well was added for neuronal patterning. On day 9, the medium was changed to the tissue growth medium, which included neurobasal medium, N2 supplement, B27 without vitamin A, GlutaMAX, MEM NEAA, and β-mercaptoethanol, supplemented with insulin, laminin, SHH-C25II, FGF8, and GFR Matrigel, for 24 hours. On day 10, the organoids were transferred to ultralow-attachment 24-well plates (3473, Corning) filled with 500 μl of organoid medium per well, containing neurobasal medium, N2 supplement, B27 without vitamin A, GlutaMAX, MEM NEAA, and β-mercaptoethanol, supplemented with brain-derived neurotrophic factor, glial cell line–derived neurotrophic factor, ascorbic acid, dibutyryl cyclic adenosine monophosphate (db-cAMP), and GFR Matrigel. From day 10, the medium was replaced every 3 days.

### Immunohistochemistry and image acquisition

Immunohistochemistry and image acquisition were performed on the basis of our previous study (*2*). Briefly, the organoids were immersed in 4% paraformaldehyde at 4°C overnight, washed, immersed in 30% sucrose at 4°C for 48 to 72 hours, and then moved to a cryomold and frozen in FSC 22 compound (3801480, Leica). The frozen organoids were cryosectioned, and the tissue slices were washed and permeabilized using 0.3% Triton X-100 (X100, Merck) in phosphate-buffered saline (PBS) for 30 min at room temperature. Sectioned slices were blocked using 5% normal horse serum (S-2000, Vector Laboratories) in PBS for 1 hour at room temperature. The first antibodies were diluted in the same solution and applied at 4°C overnight. The secondary antibodies were diluted at a 1:500 ratio in 3% bovine serum albumin in PBS and incubated for 1 hour at room temperature. Last, the slices were washed, stained with 4′,6-diamidino-2-phenylindole (DAPI), and mounted on slides. The slices were mounted on coverslips (0101222, Marienfeld), and the following antibodies were used: anti-NeuN (anti-neuronal nuclear protein, 1:500; 24307T, Cell Signaling Technology), anti-MAP2 (anti-microtubule-associated protein 2, 1:500; ab254143, Abcam), anti–DAT-Nt (anti-dopamine transporter antibody, N-terminus, 1:500; MAB369, Sigma-Aldrich), anti-GIRK2 (anti-G-Protein-Gated potassium channels, Kir3.2) (1:500; APC-006, Alomone Labs), anti-SOX10 (1:500; AF2864, R&D Systems), anti-rat immunoglobulin G (IgG) 488 (1:500; A11006; Thermo Fisher Scientific), anti-rabbit IgG 594 (1:500; A11037, Thermo Fisher Scientific), anti-mouse IgG 647 (1:500; A31571; Thermo Fisher Scientific), and DAPI (1:5000; D9542, Sigma-Aldrich). For histological visualization of neuromelanin granules, Fontana-Masson staining was performed by using a commercial kit (Abcam, ab150669) according to the manufacturer’s instructions. The images were acquired using the Leica Thunder DMi8 microscope (Leica) (*59*) with Lightening or Thunder LVCC (large volume computational clearing) mode at the maximum resolution. Imaris software (Oxford Instruments) was used for cell counting and visualization.
