# Supplementary material for: Axially multifocal metalens for 3D volumetric photoacoustic imaging of neuromelanin in live brain organoid
Source: Sci Adv. 2025 Jan 15;11(3):eadr0654. doi: 10.1126/sciadv.adr0654 (PMC11734735; doi:10.1126/sciadv.adr0654)
Supplement: Supplementary file 1 — Figs. S1 to S22 Legends for movies S1 and S2 [file sciadv.adr0654_sm.pdf]

Science Advances
AAAS

Supplementary Materials for

Axially multifocal metalens for 3D volumetric photoacoustic imaging of neuromelanin in live brain organoid

Aleksandr Barulin *et al.*

Corresponding author: Jong-Chan Park, jongchan@g.skku.edu; Junsuk Rho, jsrho@postech.ac.kr; Byullee Park, byullee@skku.edu; Inki Kim, inki.kim@skku.edu

Sci. Adv. **11**, eadr0654 (2025)
DOI: 10.1126/sciadv.adr0654

The PDF file includes:

Figs. S1 to S22
Legends for movies S1 and S2

Other Supplementary Material for this manuscript includes the following:

Movies S1 and S2

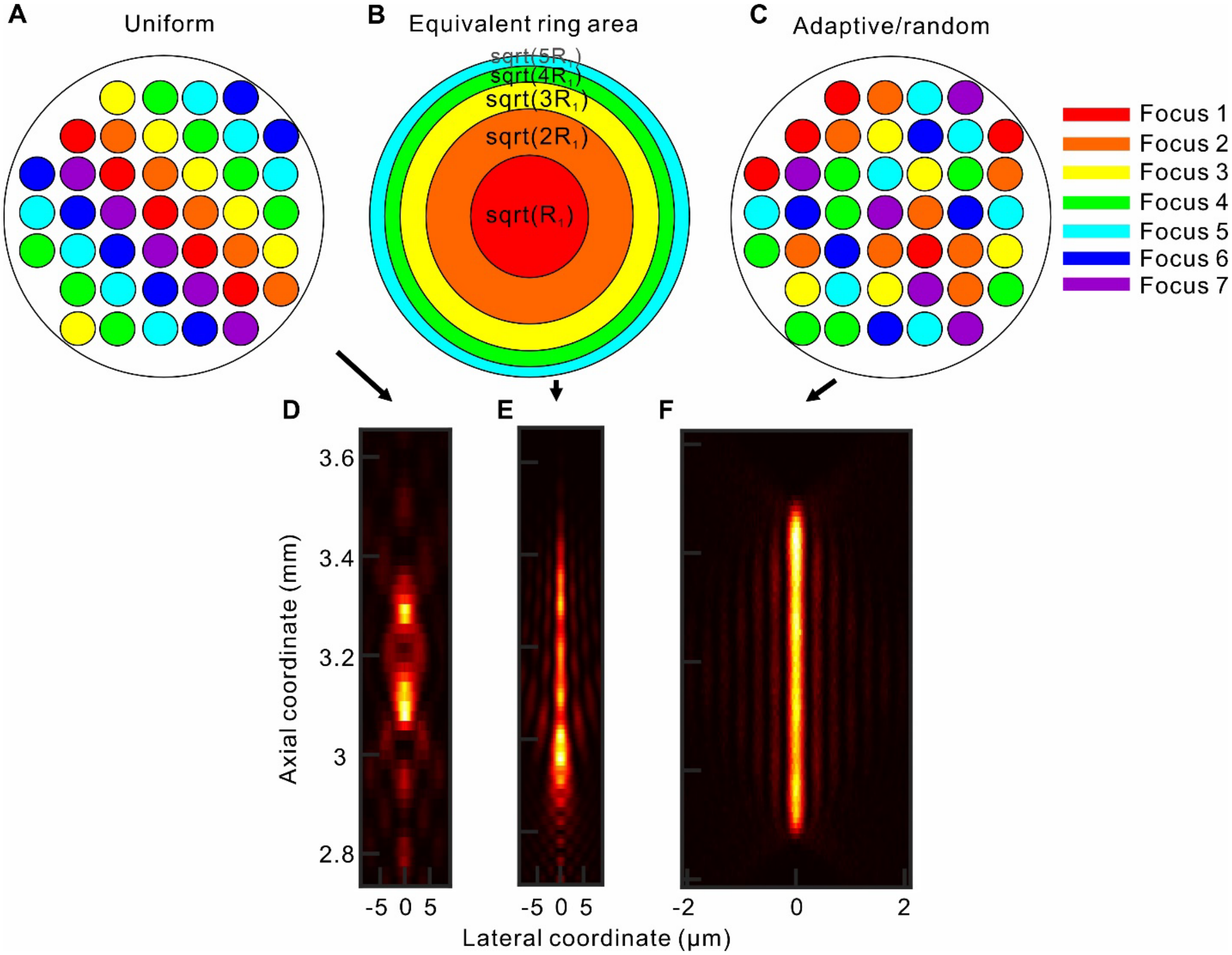

Fig. S1. Delegation of meta-atoms in the metalens lattice to focus numbers. (A) Uniform distribution of meta-atoms at foci. **(B)** Equivalent ring area distributions of meta-atoms within the metalens. The metalens area corresponding to each focus is identical and constrained within rings of outer radius $\sqrt{k \cdot R_1}$, where k is the focus number and R_1 is the radius of the first focus ring. **(C)** Adaptive or random distribution of meta-atoms at foci. Colors correspond to focus numbers, which are limited to 7 for the sake of clear representation. Axial intensity profiles for multiple foci distributed along 600 μm length for **(D)** uniform meta-atom delegation, **(E)** equivalent-ring-area meta-atom delegation, and **(F)** adaptive/random meta-atom delegation.

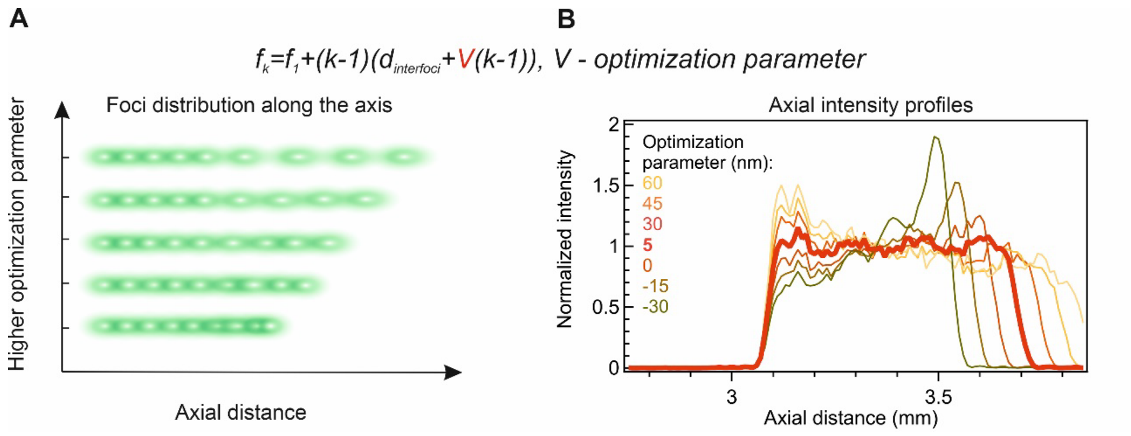

Fig. S2. Focus allocation positions for uniform axial field profile. (A) Schematic representation of foci redistribution to modify the field intensity profile through the axial beam length. Optimization parameter *V* affects the foci spreading from each other with increasing focus numbers. **(B)** Axial intensity profile for multifocal beams obtained with 60 foci and different *V* parameters.

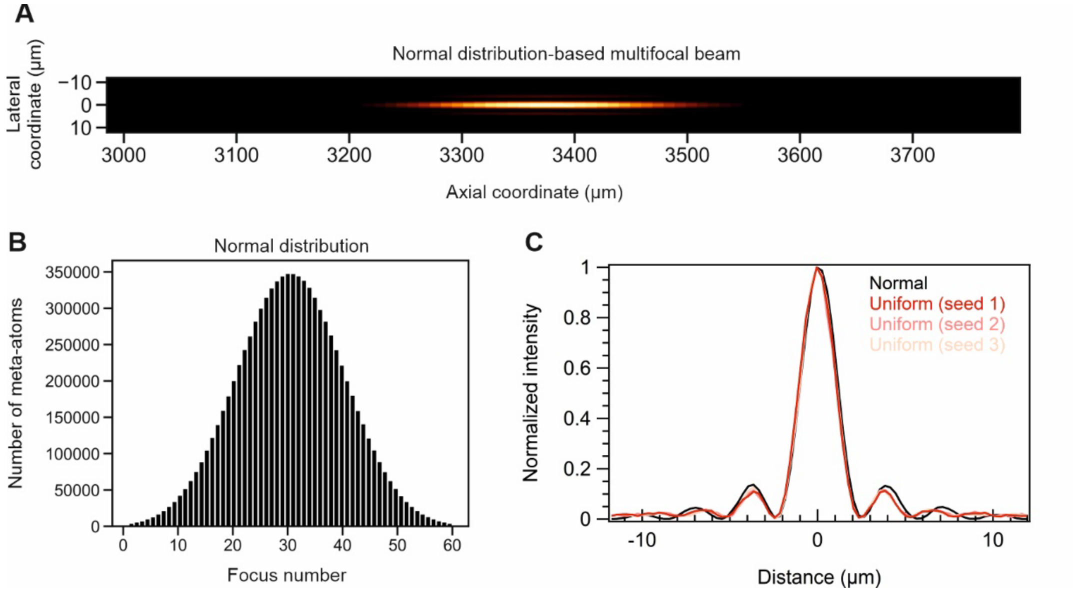

Fig. S3. Randomization influence on point spread function (PSF). (A) Axial intensity profile of multifocal metalens with normal meta-atom distribution with respect to focus numbers. The corresponding probability distribution of foci allocation is depicted on **(B)**, where 30 is the mean number, and 10 is the standard deviation, which leads to accommodation of all 60 foci within the 3σ range. **(C)** Lateral intensity profile of the multifocal beam generated via randomization based on the normal distribution from **(B)** and uniform distributions of different fixed random functions. The random functions are fixed in Python (Numpy library) via setting various random state seed values.

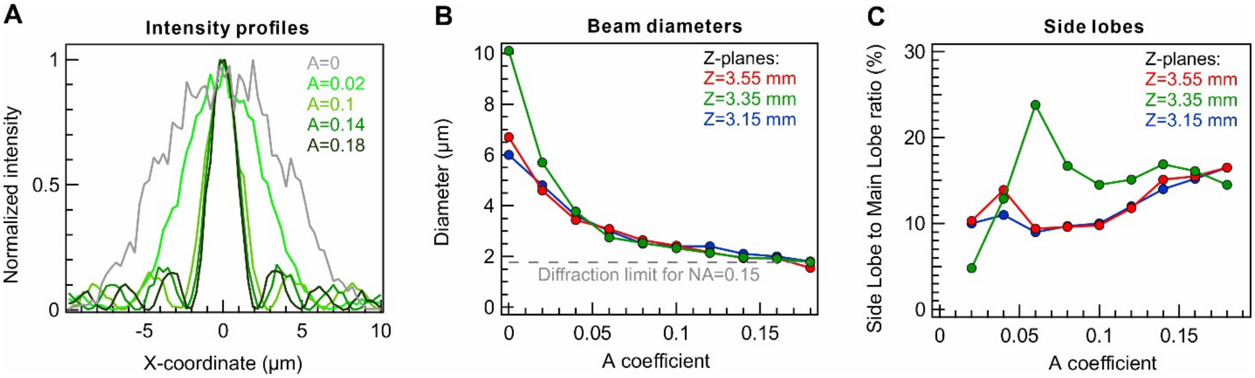

Fig. S4. Simulation of *A* coefficient influence on imaging performance. (A) Normalized lateral intensity profiles at the middle of the needle beam ($Z = 3.35$ mm) at different *A* coefficient values. **(B)** Beam diameters at three different planes ($Z = 3.15$ mm, $Z = 3.35$ mm, $Z = 3.55$ mm) determined as *FWHM* of intensity profiles. The dashed line corresponds to the diffraction-limited beam diameter of a conventional lens with NA = 0.15. **(C)** Ratio of side-lobe intensities to main-lobe intensities at three different planes ($Z = 3.15$ mm, $Z = 3.35$ mm, $Z = 3.55$ mm).

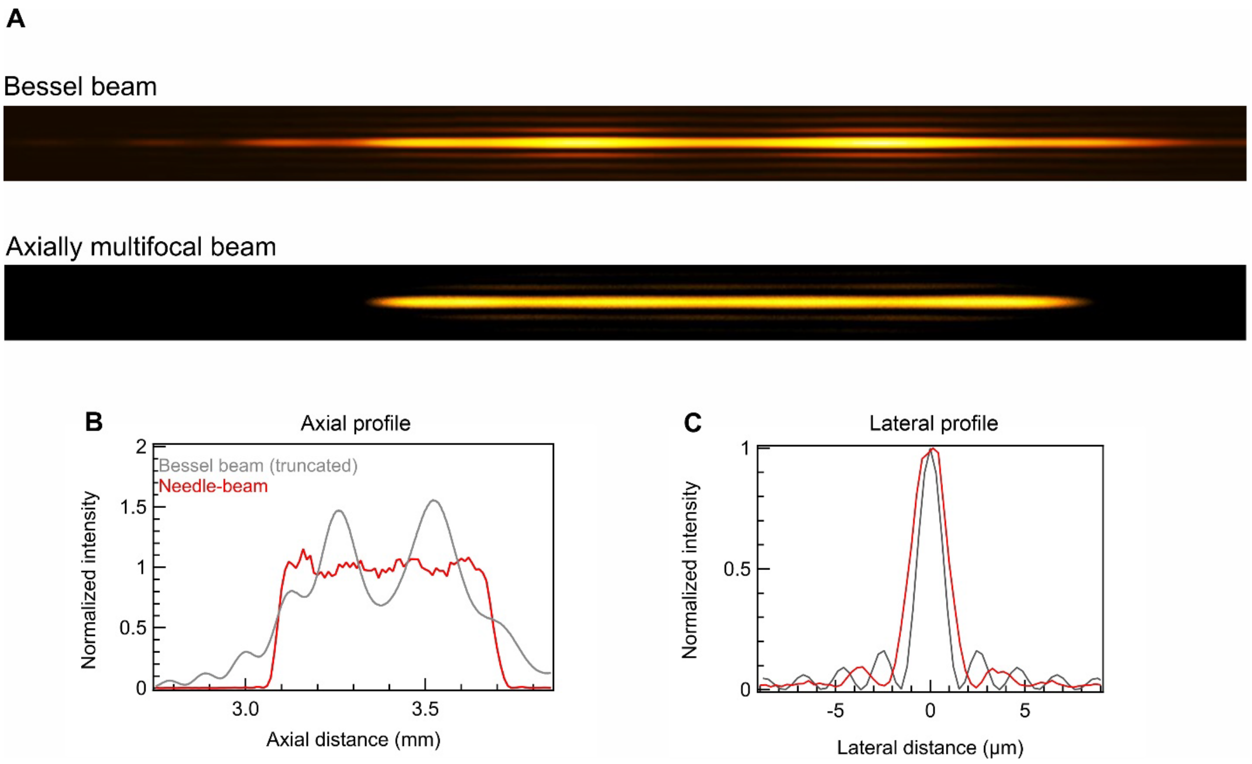

Fig. S5. Bessel beam and multifocal beam intensity profiles. (A) Bessel beam profile and axially multifocal beam profile. The Bessel beam is generated by phase-modulating annular aperture with the similar *NA* and axial beam length as the multifocal beam. **(B)** Axial intensity profiles of the Bessel beam and multifocal beam. **(C)** Lateral intensity profiles with smallest side lobes of the Bessel beam and multifocal beam.

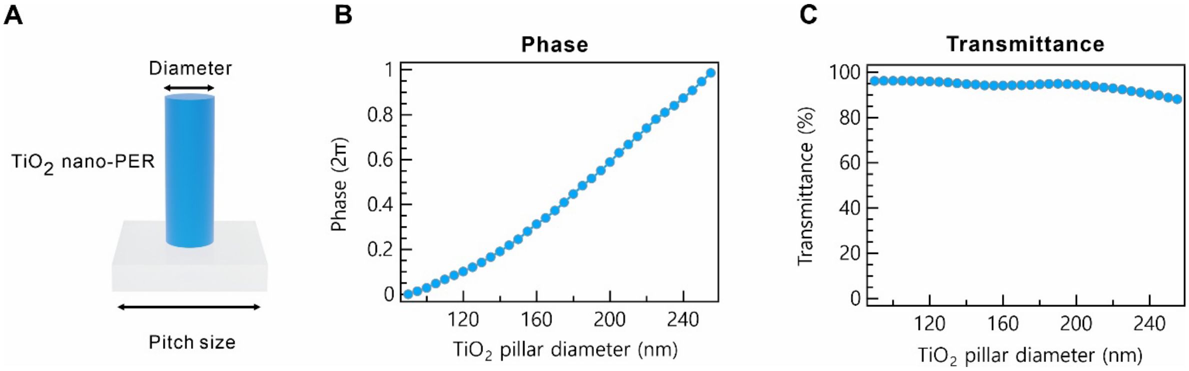

Fig. S6. Phase and transmittance of meta-atoms. (A) Meta-atom nanopillar geometry. **(B)** Phase shift of the transmitted light as a function of the nanopillar diameter. Phase value offset at 90 nm diameter of nanopillar is set to zero for demonstration of full 2π control over phase. **(C)** Transmittance as a function of the nanopillar diameter.

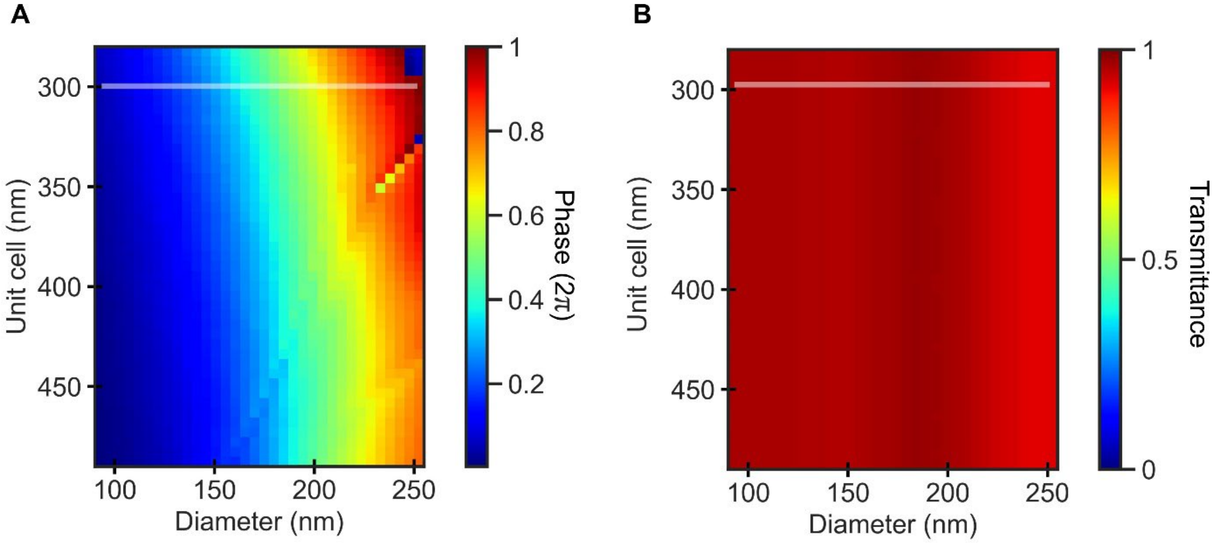

Fig. S7. Phase and transmittance versus unit cell and nanopillar diameter. (A) Map of phase dependence on unit cell size and diameter of nanopillars. **(B)** Map of transmittance dependence on unit cell size and diameter of nanopillars. The semi-transparent white line indicates the selected nanopillar geometries for meta-atom library.

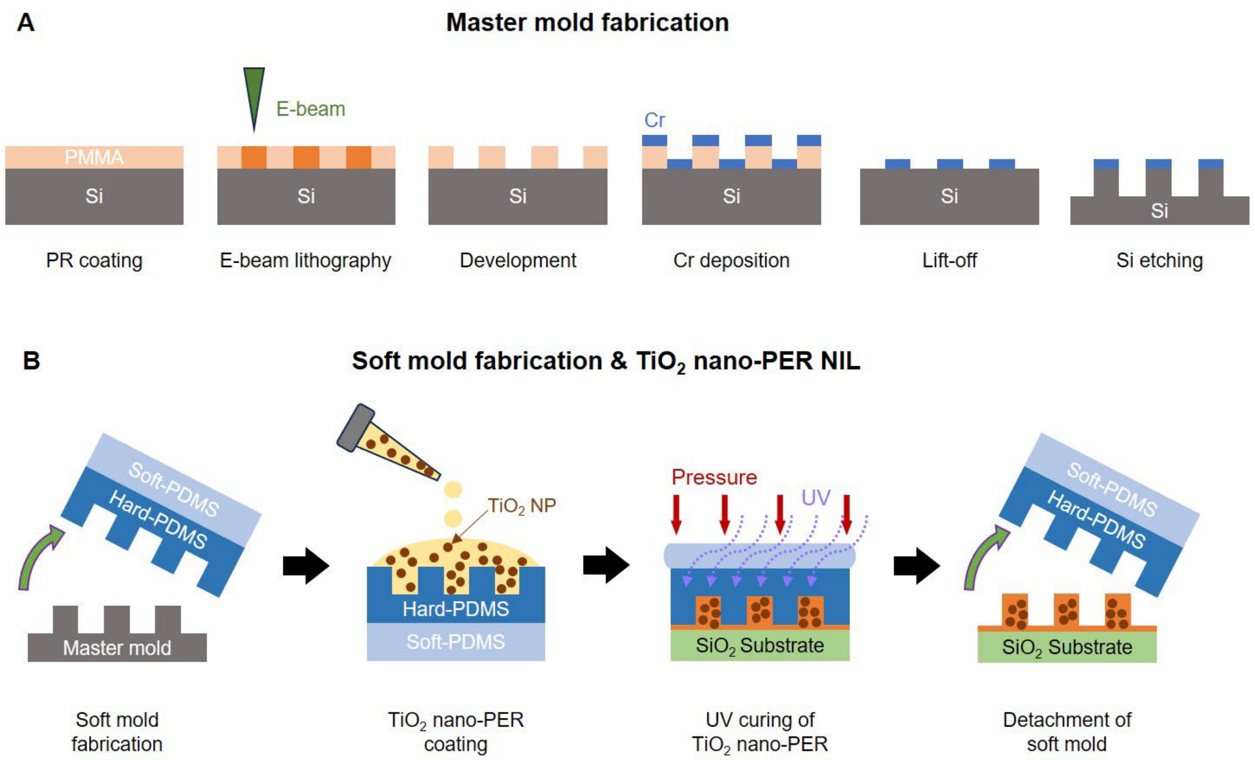

Fig. S8. Fabrication process of the axially multifocal metalens. The fabrication involves the preparation of **(A)** master mold and **(B)** soft mold, curing the titanium-oxide-nanoparticle–containing resin, and soft-mold removal.

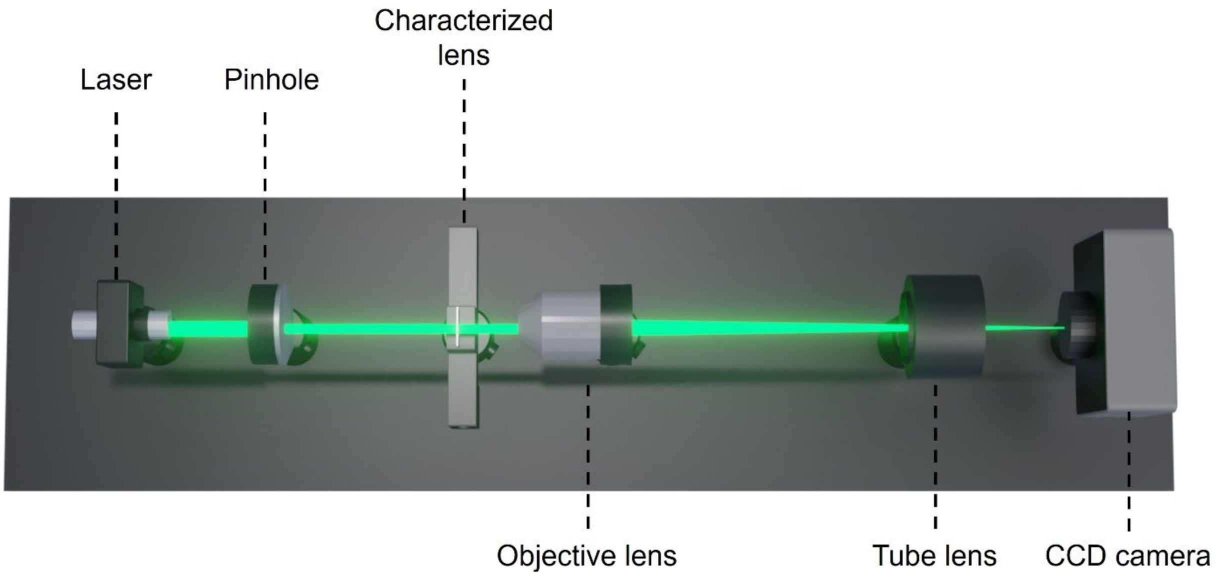

Fig. S9. Optical microscope setup for lens point spread function characterization.

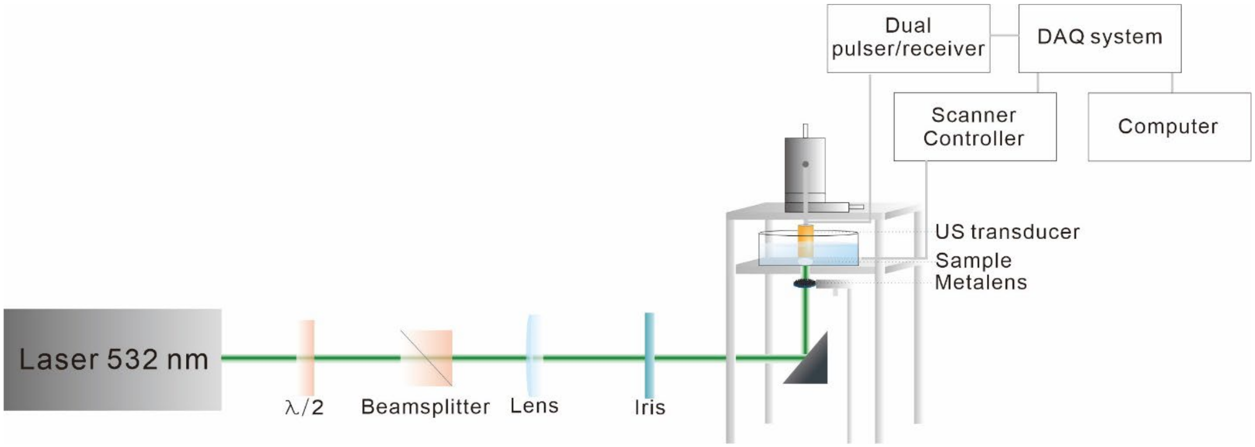

Fig. S10. Detailed experimental photoacoustic imaging system. US: ultrasound, *λ/2*: half-wave plate, DAQ: data acquisition system.

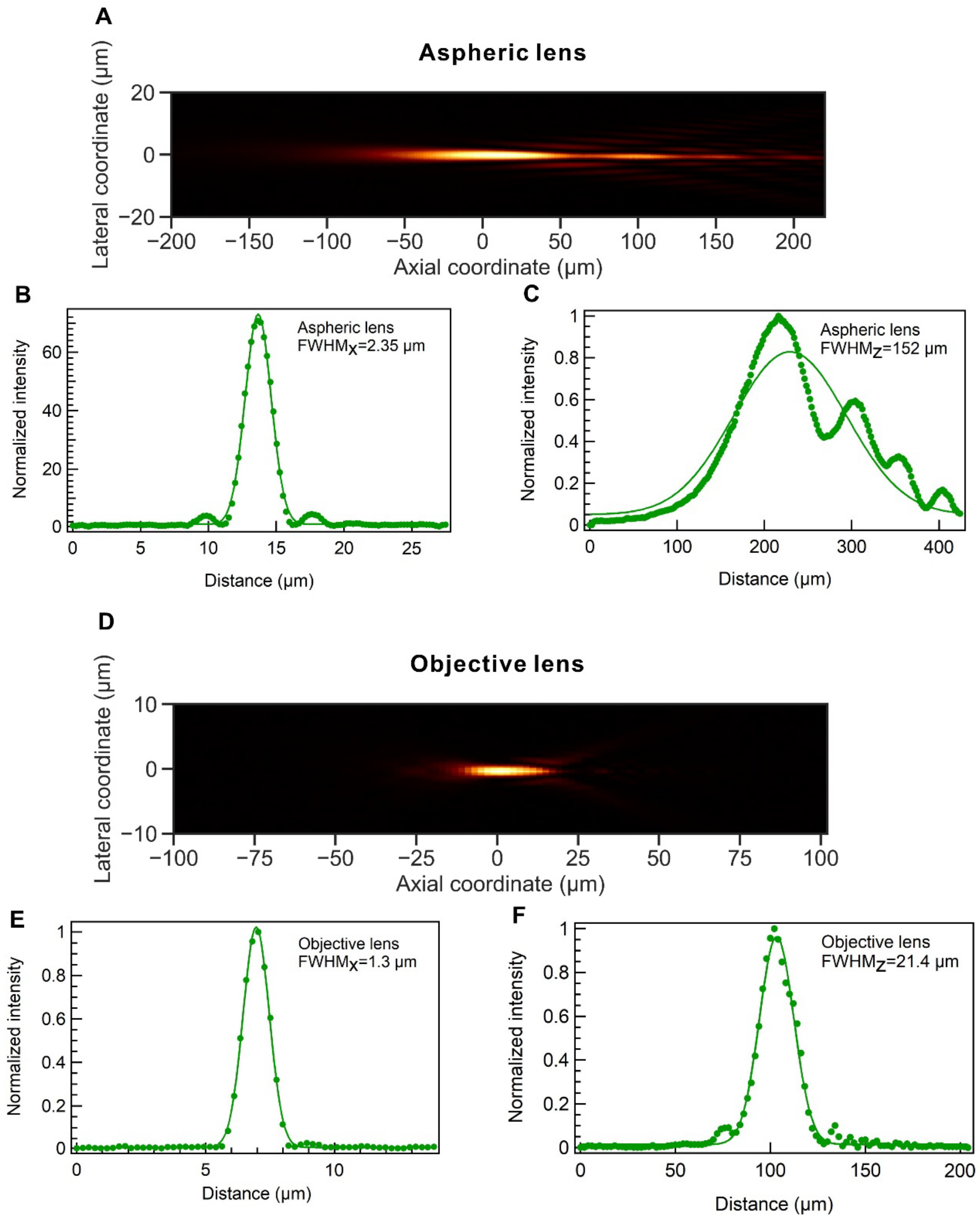

Fig. S11. PSF of conventional lenses. (A) Axial intensity profile image of the aspheric lens. **(B)** Vertical cut at zero axial coordinate of the intensity profile image of panel **(A)**. **(C)** Horizontal cut along the optical axis of the intensity profile image of panel **(A)**. **(D)** Axial intensity profile image of the objective lens. **(E)** Vertical cut at zero axial coordinate of the intensity profile image of panel **(D)**. **(F)** Horizontal cut along the optical axis of the intensity profile image of panel **(D)**.

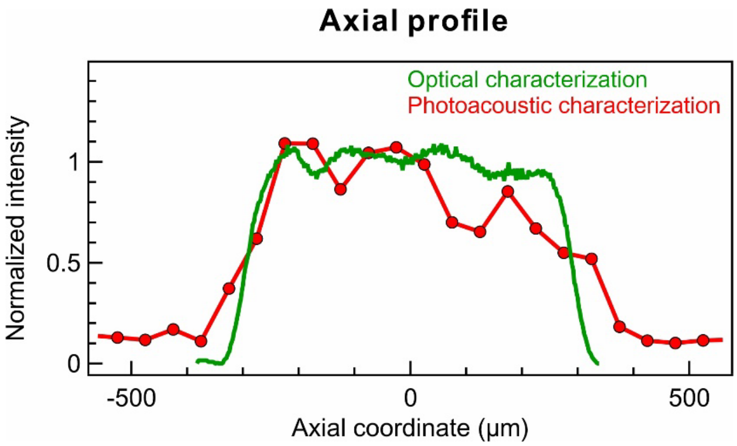

Fig. S12. Optical and photoacoustic characterization of needle-beam depth of field. Optical characterization was performed by measuring the image-based point-spread-function at multiple planes. Photoacoustic (PA) characterization was performed via carbon-fiber PA signal measurement at multiple planes.

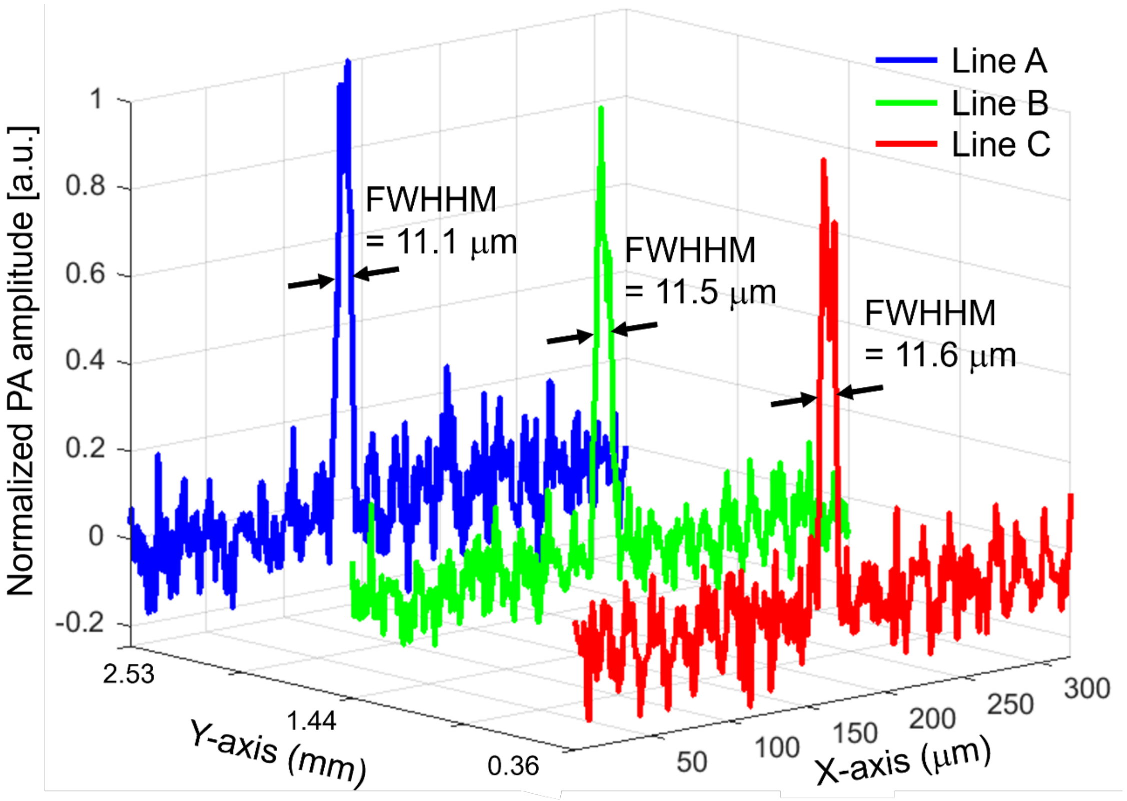

Fig. S13. Lateral line profiles and their full width at half maximum obtained at different depths of the carbon fiber sample. The full width at half maximum of the three-line profiles is 11.5 ± 0.27 μm, which aligns well with the known thickness of the carbon fiber, approximately 5 to 10 μm.

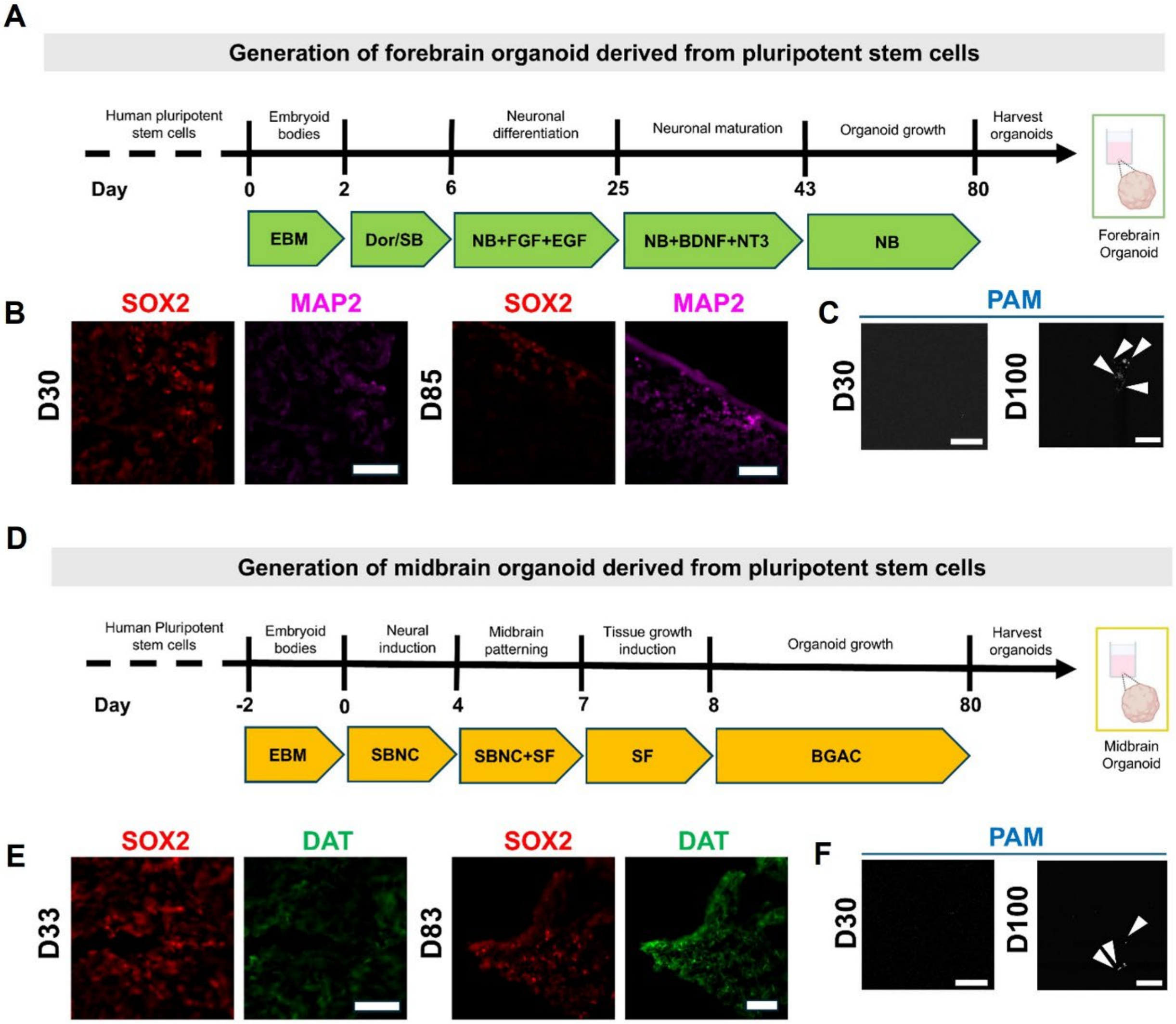

Fig. S14. Generation of brain organoids. (A) Protocol for the generation of forebrain organoids. **(B)** Immunostaining for SOX2 (neural progenitor cell marker) and MAP2 (mature neuron) at day 30 (immature) and day 85 (mature). Scale bars, 50 µm. **(C)** Photoacoustic images for neuromelanin at day 30 and day 100 for midbrain. Scale bar, 500 µm. White arrows indicate positive signals. **(D)** Protocol for the generation of midbrain organoids. **(E)** Immunostaining for SOX2 (neural progenitor cell marker) and DAT (mature dopaminergic neuron) at day 33 (immature) and day 83 (mature). Scale bars, 50 µm. **(F)** Photoacoustic images for neuromelanin at day 30 and day 100 for midbrain. Scale bar, 500 µm. White arrows indicate positive signals.

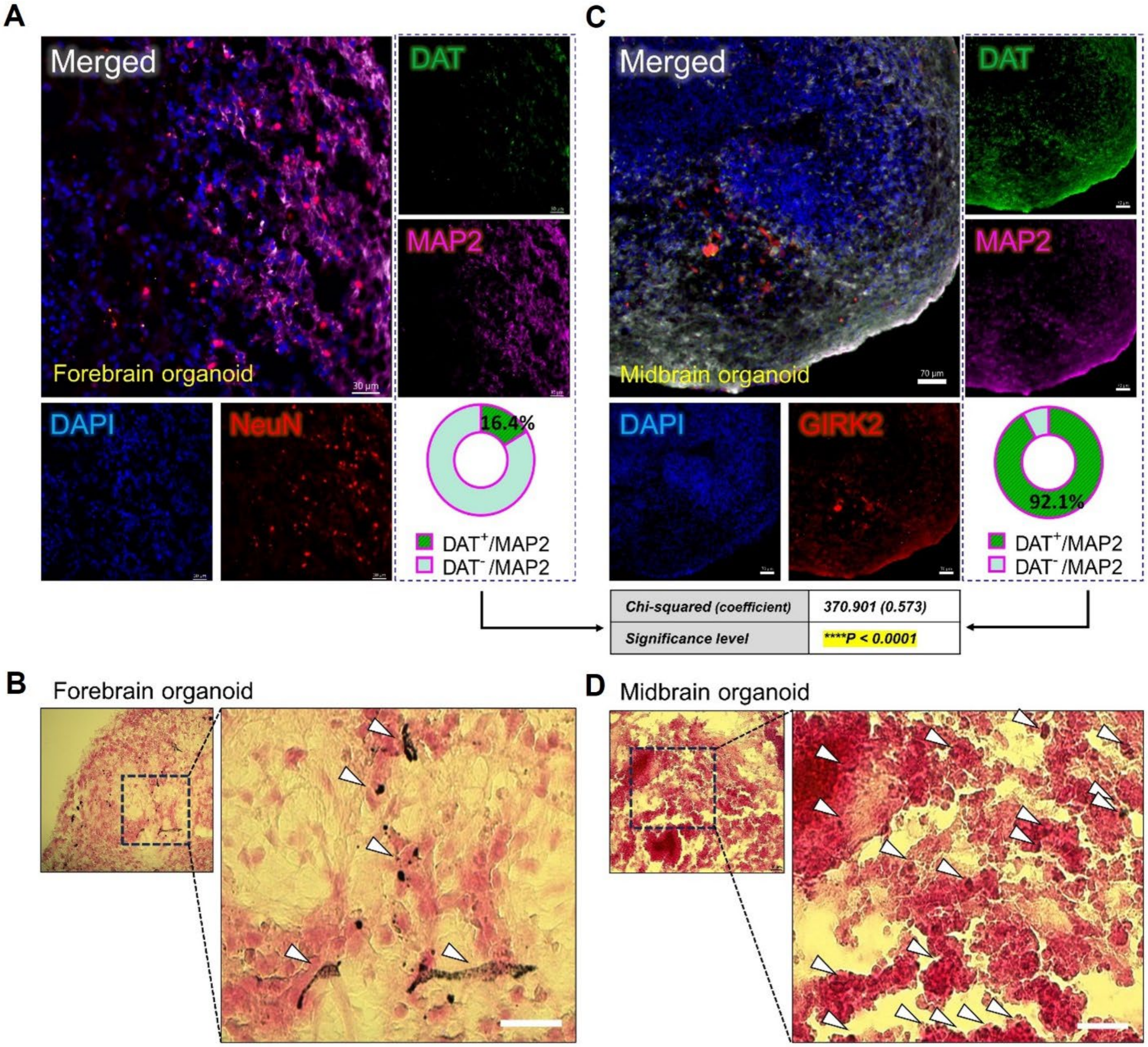

Fig. S15. Histological staining for brain organoid. **(A)** Immunostaining for the validation of neuronal gene expressions in the forebrain organoid. Dopaminergic neurons (DAT$^+$/MAP2$^+$) are partially expressed among the mature neurons (MAP2$^+$). Scale bars, 30 µm. **(B)** Fontana–Masson staining for the validation of neuromelanin (black pigments) in the forebrain organoid. White arrows indicate neuromelanin signals. Scale bar, 30 µm. **(C)** Immunostaining for the validation of neuronal gene expressions in the midbrain organoid. Dopaminergic neurons (DAT$^+$/MAP2$^+$) are mainly expressed among the mature neurons (MAP2$^+$). Scale bars, 70 µm. **(D)** Fontana–Masson staining for the validation of neuromelanin (black pigments) in the midbrain organoid. White arrows indicate neuromelanin signals. Scale bar, 30 µm. For **(A)** and **(C)**, the following antibodies and reagents were used: anti-NeuN (1:500; 24307T, Cell Signaling Technology; for neuronal

nuclear antigens; red for **(A)**), anti-MAP2 (1:500; ab254143, abcam; for mature neuronal microtubules; magenta), anti-DAT-Nt (1:500; MAB369, Sigma-Aldrich; for partially expressed dopaminergic neurons; green), anti-GIRK2 (1:500; APC-006, Alomone lab; for potassium channels in dopaminergic neurons; red for **(C)**), DAPI (1:5000; D9542, Sigma Aldrich; for cellular nucleus; blue). The images were acquired using the Leica Thunder DMi8 microscope (Leica). The P-value was obtained via a Chi-square test, $****p < 0.0001$. The zoomed-in images of **(B)** and **(D)** are used for Fig. 4F and 4L.

Abbreviations: NeuN, neuronal nuclear protein; MAP2, microtubule-associated protein 2; DAT, dopamine transporter; GIRK2, G-protein-activated inward rectifying potassium type 2; DAPI, 4′,6-diamidino-2-phenylindole.

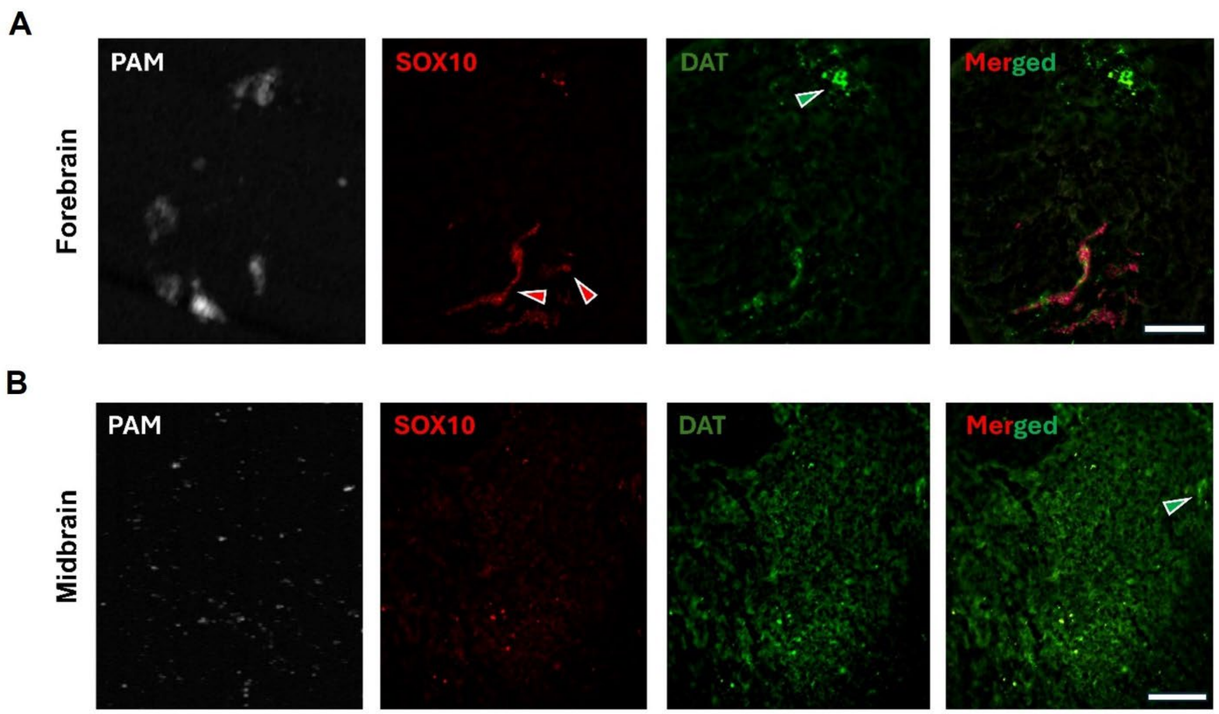

Fig. S16. Neuromelanin production. (A) Melanin production via both neural crest cells ($SOX10^{+}$) and dopaminergic neurons (DAT^{+}) in the melanin-containing forebrain organoids. Scale bar, 50 µm. **(B)** Melanin production via both neural crest cells ($SOX10^{+}$) and dopaminergic neurons (DAT^{+}) in the midbrain organoids. Red arrows, $SOX10^{+}DAT^{-}$; green arrows show $SOX10^{-}DAT^{+}$ signals; Scale bar, 100 µm.

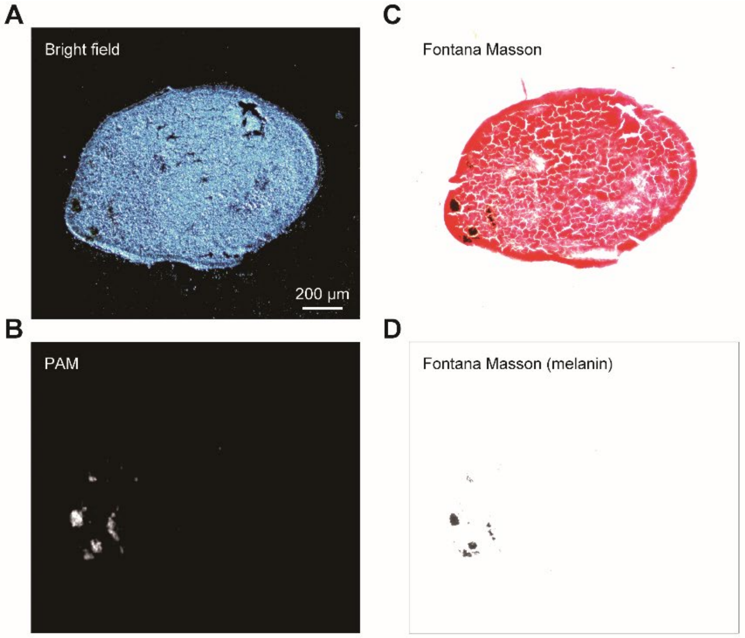

Fig. S17. Correlative imaging of PA and Fontana-Masson staining. (A) Bright field image of sectioned organoid. **(B)** PA maximum amplitude projection (PA MAP) and **(C)** Fontana-Masson image of the same organoid. **(D)** Highlighted melanin from the Fontana-Masson image.

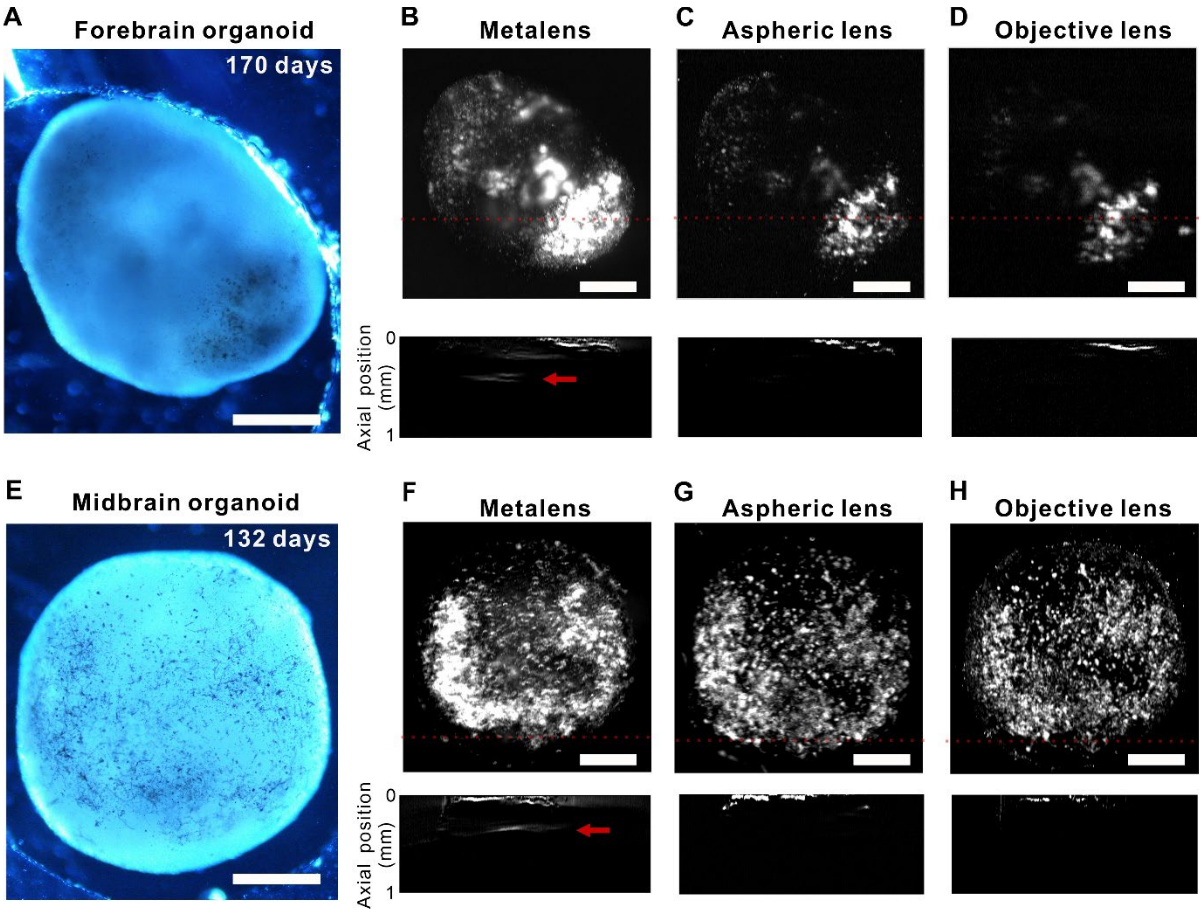

Figure S18. Bright-field and PA MAP images of brain organoids. (A) Bright field image of forebrain organoid grown for 170 days. **(B-D)** PA images of forebrain organoid with **(B)** axially multifocal metalens, **(C)** aspheric lens, and **(D)** objective lens. The panels at the bottom of **(B-D)** correspond to the axial position profiles of the PA signal along the dashed lines. **(E)** Bright field image of midbrain organoid grown for 132 days. **(F-H)** PA images of midbrain organoid images with **(F)** axially multifocal metalens, **(G)** aspheric lens, and **(H)** objective lens. The panels at the bottom of **(F-H)** correspond to the axial position profiles of the PA signal along the dashed lines. PA, photoacoustic. The red arrows show neuromelanin visualized by the metalens in the deep of the brain organoids. Scale bars, 500 μm.

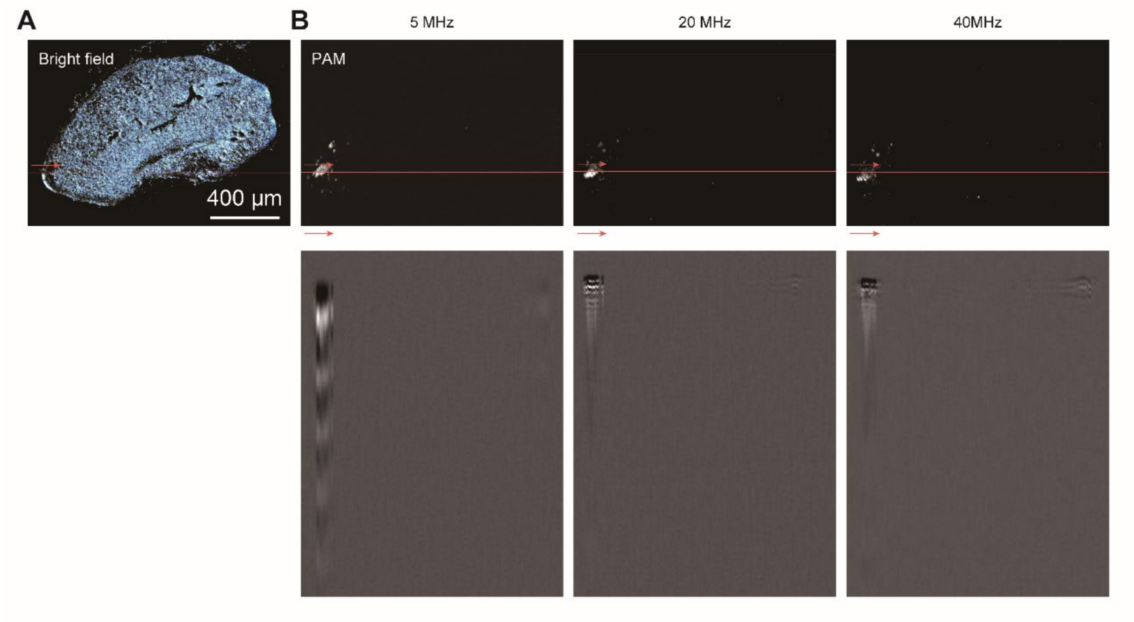

Fig. S19. Transducer frequency dependency of PA imaging. (A) Bright field image of sectioned brain organoid. **(B)** The PA MAP images of the same sectioned organoid in **(A)**, obtained using transducers with center frequencies of 5, 20, and 40 MHz. The images at the bottom are B-scan images corresponding to the red lines.

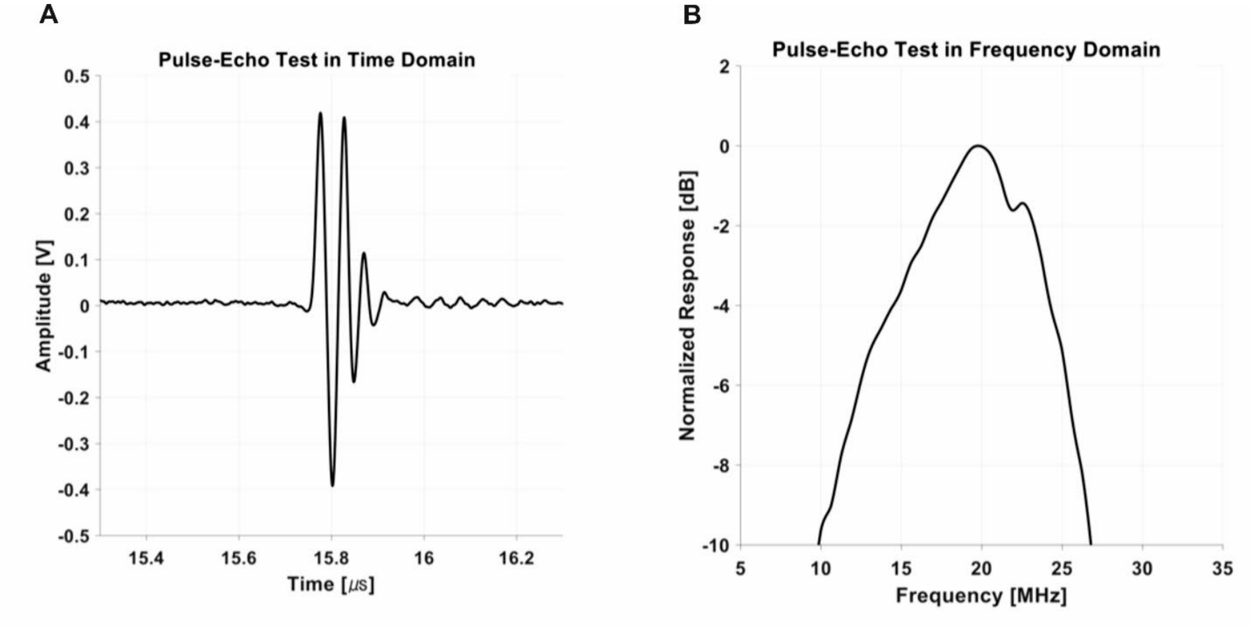

Fig. S20. Pulse-echo test of the ultrasound transducer. (A) Pulse-echo test in time domain and **(B)** in frequency domain.

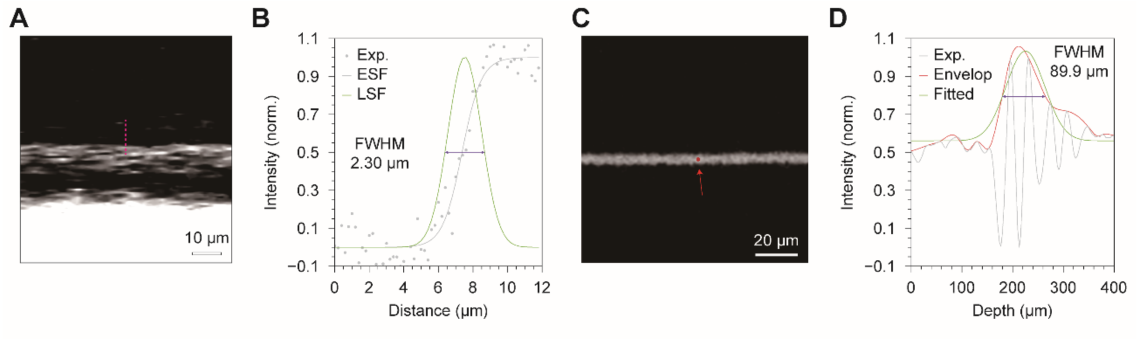

Fig. S21. Resolution of metalens-based photoacoustic imaging. (A) PA MAP image of a blade. The dashed line indicates where the edge spread function was obtained. **(B)** Edge spread function (ESF), and line spread function (LSF) of the blade. The measured lateral resolution was 2.30 µm. **(C)** PA MAP image of a carbon fiber. The arrow indicates the points where intensity profile was obtained. **(D)** Axial intensity profile of the carbon fiber and its envelope. The acquired axial resolution was 89.9 µm in axial resolution.

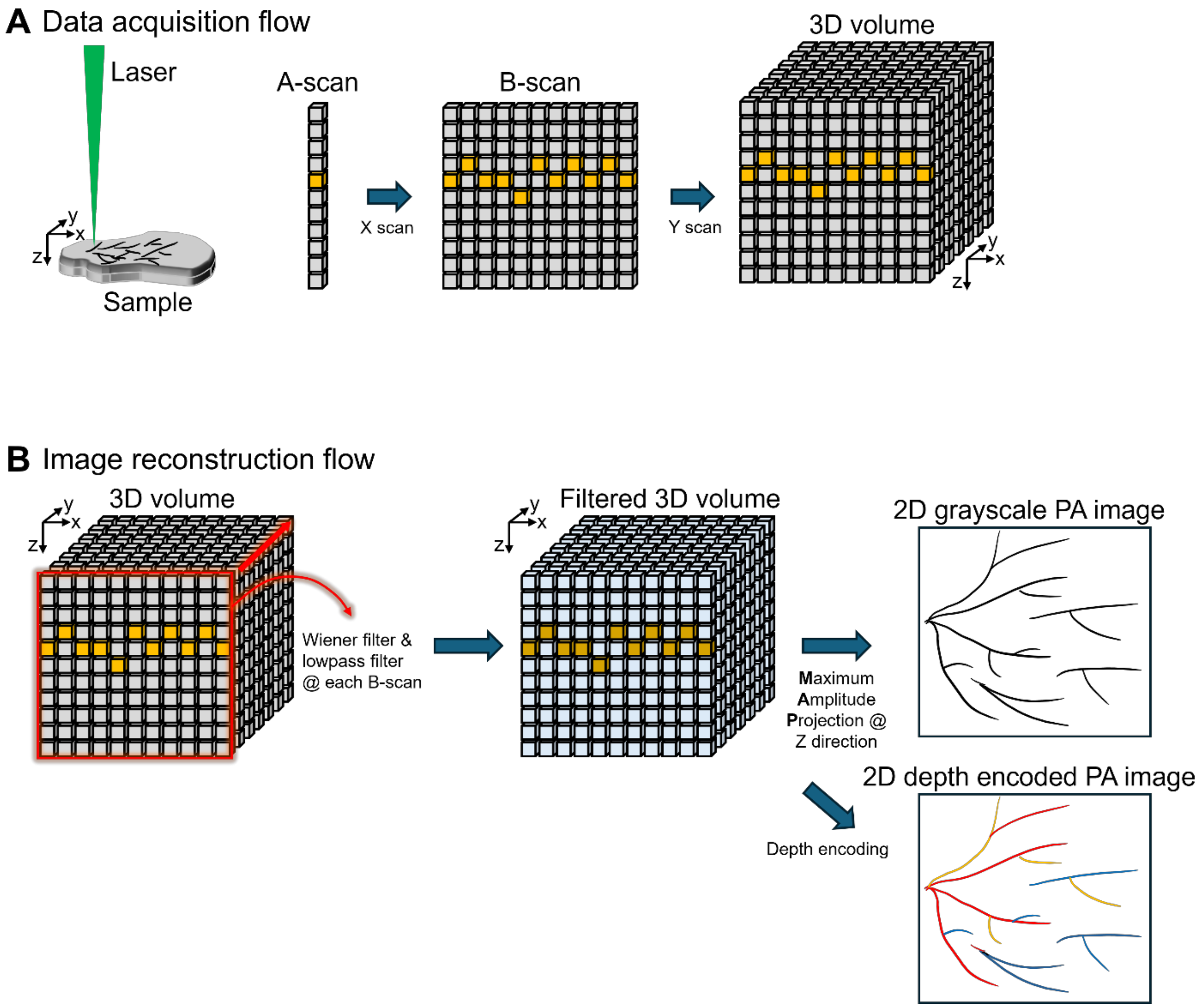

Fig. S22. Image processing of photoacoustic image. (A) Data acquisition flow and **(B)** image reconstruction flow

Movie S1. 3D volume rendering of forebrain organoid photoacoustic image

Movie S2. 3D volume rendering of midbrain organoid photoacoustic image
